# The Role of Reinforcement Learning in Pragmatic Reasoning Tasks: Modeling and Validating the Sources of Individual Differences

**DOI:** 10.1162/OPMI.a.341

**Published:** 2026-03-15

**Authors:** John Duff, Alexandra Mayn, Vera Demberg

**Affiliations:** Department of Linguistics, University of California, Los Angeles, Los Angeles, CA, USA; Department of Language Science and Technology, Saarland University, Saarbrücken, Germany; Department of Computer Science, Saarland University, Saarbrücken, Germany

**Keywords:** pragmatic reasoning, individual differences, reinforcement learning, resource-rationality, ACT-R

## Abstract

In Gricean pragmatics, inference during communication is regarded as a form of rational, domain-general reasoning about the intentions of other agents. Studies using the pictorial communication “reference game” task are sometimes used in support of this hypothesis. Yet, measures of pragmatic reasoning in this task sometimes reveal poor performance, with participants requiring many rounds of play before they exhibit patterns which match Gricean inferences, and demonstrating substantial individual differences in behavior. Do these results challenge the idea of widespread inferencing via fundamental social competence? We advance an alternative proposal here, which posits that these patterns emerge as a factor of the way participants perform pragmatic reasoning in a task: namely, they prefer to use simpler interpretation strategies until experience motivates the use of additional resources. Building off of work modeling task adaptation as reinforcement learning, we use the cognitive architecture ACT-R to simulate the expected behavior of individuals with this kind of resource-rational performance algorithm, subject to individualized parameters for reinforcement learning. These simulations provide a proof-of-concept for our adaptation proposal, recreating known patterns and generating new concrete predictions for the particular domain-general sources of individual variance in reference game tasks. We then go on to validate some of these new predictions in a pre-registered experiment, and find that pragmatic response behavior is indeed related to a participant’s general persistence in self-directed exploration of strategies for task completion. Our results offer a path to reconcile variable empirical data with models of core pragmatic competence. From a broader perspective, we see this as an important step towards more robust theories of performance factors in pragmatic reasoning, and ultimately, a case study in the value of process-level computational modeling.

## INTRODUCTION

Humans routinely use and interpret linguistic descriptions which leave out information that is needed in context. As an example, imagine you are the proprietor of a bakery that has three items on its menu: an apple pie, an apple pie with a cinnamon crumb topping, and a Black Forest cake. If a customer orders “apple pie”, in principle you might need to ask for clarification: there are two different options available which meet that literal description. But in practice, there will likely be no confusion at all, and you might just hand the customer what you understood them to ask for, a slice of the plain apple pie.

Since Grice (1975), our ability to draw such inferences has been explained as part of a general pragmatic calculus. That is, extra information about the communicative intentions of a speaker—Grice’s “conversational implicatures”—can be recovered under the assumption that the speaker is picking messages cooperatively. For instance, we expect that speakers will provide enough information to achieve their goals. This is what allows us to derive the implicature above that our customer wanted the plain pie: a cooperative speaker in this situation would have said something more if they wanted the crumb-topped pie. In particular, this is an “*ad hoc* implicature,” as it depends crucially on the set of conceivable intentions given the context. On another day, where the options are the crumb-topped apple pie, the Black Forest cake, and a cheesecake, there’s no doubt that an order of “apple pie” would now mean the one with the topping.

The overall picture of language comprehension that emerges is one where we recover literal meaning using lexical and grammatical knowledge, but then apply additional, context-specific reasoning about goal-oriented behavior to derive an enriched interpretation. Some researchers have adopted the formal tools of game theory to characterize this state of affairs as a rational equilibrium for multi-party communication (e.g., Franke, 2011; Jäger, 2010; Parikh, 1991; Van Rooy, 2004). Branching off from the game-theoretic literature, Frank and Goodman (2012) bridged these ideas with contemporary models of social cognition (Baker et al., 2009), framing communicative rationality as Bayesian inference. In their Rational Speech Act (RSA) model (see Degen, 2023; Goodman & Frank, 2016 for a review), a comprehender’s interpretive choices can be treated as rational given a probabilistic model of the speaker’s likely behavior. This architecture permits infinite recursion: that speaker model can itself be a model of a rational agent optimizing messages against some comprehender model, which can itself be a model optimizing for some speaker, etc. To capture the variety of cooperative reasoning envisioned by Grice, it is enough to consider a model containing just two levels of recursion: a “second-order” pragmatic comprehender reconstructing the likely intentions of a “first-order” pragmatic speaker, who was optimizing for a literal comprehender.

The RSA model has been especially influential through its success in accounting for behavioral data in simplified communication tasks. These tasks are often “reference games”, tasks similar to the bakery scenario above, but with pre-defined sets of possible messages, and a tightly-restricted feature geometry for possible referents. And indeed, participants in these reference games, including young children, have been widely observed to exhibit inferences sensitive to expected speaker behavior in the way expected by RSA models and their cousins (Carstensen et al., 2014; Degen & Franke, 2012; Frank et al., 2016; Frank & Goodman, 2012; Qing & Franke, 2015; Rohde et al., 2012; Stiller et al., 2011; Stiller et al., 2015). These results nicely support the idea that rational inference over recursively cooperative behavior is a general faculty that humans deploy expertly across a variety of communicative scenarios.

Nevertheless, the patterns observed in reference game studies are complex, and do not align exactly with the behavior predicted by a typical RSA model. First, comprehenders may not actually engage in second-order reasoning so readily. Sikos et al. (2021a), investigating specifically single-trial games which required second-order reasoning, found very little evidence that it was successfully applied. Second, even in multi-trial games where second-order reasoning emerges more clearly, studies comparing across participants (Franke & Degen, 2016; Mayn & Demberg, 2023) have observed a spectrum of behavior, with many individuals matching models of first-order inferencing, rational interpretation based on a model of a literal speaker. Targeted investigation of these individual differences by Mayn and Demberg (2026) found that parts of this variance are related to variation in performance on social reasoning tasks, but also participants’ performance on problem-solving tasks without social or communicative dimensions, like Raven’s Matrices (Raven et al., 1998).

An idealized model of the abstract human capacity for recursive game-theoretic reasoning can’t explain these complications on its own. On their face, it may seem that these results pose some challenge to the idea that such reasoning is a core human expertise that underlies inferences we draw frequently in daily communication.

In this paper, we will counter this challenge, showing that these patterns may in fact emerge from how comprehenders deploy a flexible capacity for Gricean reasoning. We propose a model of reference game performance where comprehenders can pursue several strategies for interpreting the messages they receive, namely strategies akin to literal, first-order, and second-order reasoning in an RSA model. Success then depends on participants’ ability to adapt to the task: they must try to identify a resource-rational interpretive strategy for the current interaction as they progress through game rounds, learning from exploration and failure. We implement this model in the cognitive architecture ACT-R (Anderson et al., 2004; Anderson & Lebiere, 1998) to investigate its quantitative predictions.

In an initial simulation, we consider the patterns of behavior generated by our ACT-R model, finding that the model can successfully reproduce the adaptation curves and individual difference patterns observed in previous experiments with human subjects. In particular, individual differences are predicted as a consequence of variation in parameters for reinforcement learning, which drives adaptation in our model. Given recent work highlighting the effects of reinforcement learning parameters in an ACT-R model for Raven’s Matrices (Stocco et al., 2021), we find this to be a plausible explanation for the correlation between reference game and Raven’s performance observed in Mayn and Demberg (2026). We then move on to report a pre-registered individual differences study with human participants, testing the key predictions of our model by collecting independent measures of two relevant adaptation parameters: the strength of negative feedback, and persistence in the search for a solution. We find that they indeed explain variation in participants’ reference game performance, especially the latter, although some aspects of the data mismatch the ACT-R model’s specific quantitative predictions, highlighting the places where our approach remains simplistic.

We conclude that effective resource-rational adaptation to the demands of a task plays a substantial role in achieving success in a reference game, and variance in adaptation behavior may explain a great deal of individual differences in these tasks. This leaves room for a core Gricean competence, but highlights that reference games do not offer an opportunity to probe it directly, only through the noisy channel of task performance.

## INFERENCE BEHAVIOR IN REFERENCE GAMES

### Game Properties

In the reference games we will focus on here, participants play as comprehenders, selecting from an array of three possible referents based on a short message. Comprehenders are told the message was chosen by another player, who was trying to help them select a specific target referent out of the three. In the present work, as in Frank and Goodman (2012), the three possible referents are simple icons, defined by the combination of shape (a circle, a square, or a triangle) and color (blue, green, or red). Messages were also icons, depicting a single one of these features, e.g., an un-filled triangle, or a spilling tube of red paint. Finally, as in Degen and Franke (2012) and Franke and Degen (2016), we will discuss games where comprehenders and fictional speakers were jointly aware of an additional constraint on the speaker: they could only select from a bank of four possible messages, leaving two of the six feature values inexpressible. In our studies, it was never possible to communicate the color blue or the shape square.

**Figure 1.**
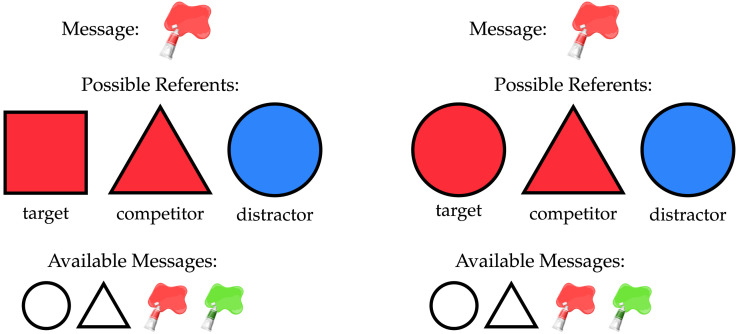
An example “simple” critical trial (left) and an example “complex” critical trial (right).

Within this setup, we can follow Stiller et al. (2011) and Degen and Franke (2012) to distinguish two categories of “critical” game trial, where the literal meaning of the given message was not sufficient to identify the target, but Gricean inferences can provide a solution. The left side of Figure 1 demonstrates a “simple” critical trial. In “simple” critical trials, two of the three possible referents have a feature matching the given message, but these two referents differ in whether they have a second feature which can also be expressed using the message bank. One possible referent (here, the red triangle) has an alternative message available, and for the other (here, the red square), the observed message would be the only option. A comprehender can draw an inference by comparing the expected behavior of a rational speaker describing these two possible referents. In a model of second-order reasoning for the comprehender, they should expect the speaker to select messages which maximize the chance that a literal comprehender could pick correctly. With that literal comprehender, for the red triangle, the message “red” will only succeed in 50% of rounds, as they will have to guess randomly between the red triangle and the red square. In contrast, for the red triangle the message “triangle” will succeed in 100% of rounds. A speaker taking this into consideration would then favor “triangle” in the case of that referent, while they can only say “red” for the red square, given the constraints of the message bank. A comprehender considering the speaker’s likely behavior would thus favor the hypothesis that the speaker was describing the red square, as that is the case where this message was most likely to be observed. In describing these game scenarios, we will call the referent favored by Gricean inference the “target.” The other possible referent which matches the observed message will be the “competitor,” and the final non-matching referent will be the “distractor.”^1^

As noted by Degen and Franke (2012), in game scenarios like this one, a preference for the target can also emerge from a simpler first-order model of the comprehender, limited to reasoning about a literal speaker. This is because even a literal speaker, who selects randomly from any matching messages from the message bank, will be more likely to use the ambiguous “red” message when describing the red square (100%) than the red triangle (50%), just because of the number of available messages. This scenario is in fact not limited to game setups with artificially constrained message banks. In a different feature geometry used first by Stiller et al. (2011), based on faces with various accessories (glasses, hats), the number of alternative messages may differ between two objects in the same way whenever only one object possesses a second feature to be communicated. Indeed, this is the situation in our “apple pie” hypothetical from the introduction. As Franke (2011) observes, this can also be mapped to the very salient real-world case of scalar implicature, where asymmetric entailments between two terms describing values on the same scale (all “hot” things are “warm” but not vice versa) guarantee that some possible states of affairs have more alternative descriptions than others. First-order recursion is sufficient, then, to account for target preferences in any of these contexts; this data on its own does not provide clear evidence for second-order recursion, the sophisticated consideration of a *cooperative* speaker that underlies a standard Gricean pragmatics.

The right side of Figure 1 demonstrates a “complex” critical trial. Like the case above, two of the three possible referents have a feature matching the given message, but unlike the case above, both also have a second feature which can also be expressed using the message bank. As a literal speaker would use the ambiguous message “red” equally for both referents, a first-order model of comprehension would not be able to derive any preference here. What makes a solution possible at all in this scenario is a difference of utility between the alternative messages for each possible referent, which will only be exploited by a first-order speaker, and thereby will only be considered by a second-order comprehender. One of the two matching referents has an alternative message which is unambiguous in this context: a literal comprehender hearing the message “triangle” would always select the red triangle. A speaker reasoning about such a listener will then favor “triangle,” and disfavor “red,” for that referent. This produces a critical asymmetry in the use of “red”: it will be more frequent when describing the red circle, where it is not overshadowed by a better message. A comprehender reasoning about such a speaker would then favor the red circle when “red” is the message.

The patterns of possible success and failure we have laid out can be derived in the Bayesian approach of RSA models, as well as closely-related, more deterministic alternatives like the iterated best response models of Jäger (2010) and Franke (2011). One place where the two types of accounts differ is in exactly how the expected reasoning of a simulated partner informs the reasoning at the next level up. While iterated best response models expect all agents to capitalize fully on any asymmetry, always selecting the “best response,” RSA models treat all agents as probabilistic, so that uncertainty over a partner’s expected reasoning may feed forward to non-deterministic behavior at the next level up.^2^ RSA predictions were thus originally tested in reference games by probing uncertainty over responses somewhat directly through betting paradigms (Frank & Goodman, 2012), although it has become more typical to use choice probabilities across many trials to calculate uncertainty indirectly (Frank et al., 2016; Qing & Franke, 2015; Sikos et al., 2021a).

The predictions of RSA models may in fact differ from the intuitive patterns above, as they do not solely model speaker and comprehender behavior as dependent on the abstract properties of the game. Agents are also expected to take into account prior probabilities. Specifically, in the second-order comprehension model as presented in Frank and Goodman (2012), the comprehender’s final selection probabilities are the product of the likelihood derived from expected speaker behavior, and a prior probability over referents. This prior is cast intuitively as a salience metric, a distribution over what referent a speaker would be most likely to refer to, absent any knowledge of the message. On any given trial, the prior can have a strong effect on participants’ final selections, sometimes overwhelming any smaller preferences contributed by the likelihood. This prior-dependence is well-motivated as a part of rational behavior, but as we will see, it competes with recursive complexity as a source of predictive power in RSA models.

### Second-Order Reasoning in Single-Round Games

Frank and Goodman (2012) initially validated second-order RSA models as explanations for comprehension by testing the correlation between model predictions and observed bets in a sample of single-round reference games. RSA model predictions were calculated from empirical foundations, estimating likelihoods based on responses from other participants playing as speakers, and estimating priors based on responses from other participants playing as comprehenders who received an unintelligible message, thought to measure something akin to visual salience. The predictions that the RSA model derives based on these measures correlated remarkably well with the observed bets from players playing as comprehenders (*r* = 0.99). Nevertheless, the game scenarios used for this experiment were sampled equally from six categories over the space of possible game scenarios, defined in terms of feature alignment among the target and other possible referents, which were two-feature icons as above. This had the consequence, as highlighted in an in-depth critique by Sikos et al. (2021a), that only one out of twelve of the game scenarios instantiated a complex trial as described above. The games had an unconstrained message bank, and all referents always matched two possible messages, so there were no cases of simple trials. As a result, on the remaining eleven out of twelve of the possible trials shown, either messages were unambiguous, and therefore variance in the predictions was trivial, or likelihoods would be expected to be equal across multiple referents, and therefore variance in the predictions was influenced by the prior alone.

In fact, when Sikos et al. (2021a) collected new data in the same distribution of game trials, they found that high correlations were attributable not to RSA models’ ability to capture Gricean reasoning in the few complicated trials, but instead to their sensitivity to asymmetric priors in the remaining cases. Across the whole sample, a second-order RSA model did not provide a significantly better fit than a literal comprehender model enriched with the same empirical salience priors. The problem seems to be that complex reference game trials in short studies are simply not solved in the way expected above. Indeed, when isolating complex trials, performance is close to chance (Frank et al., 2016; Stiller et al., 2011), at least for certain types of message (Qing & Franke, 2015; Sikos et al., 2021a). In comparison, most participants recover target interpretations in simple trials in short studies (Frank et al., 2016; Stiller et al., 2015).

There is stronger evidence for Gricean interpretations in complex trials when participants played extended multi-round games, largely finding average target choices slightly but significantly above chance (Degen & Franke, 2012; Franke & Degen, 2016; Mayn & Demberg, 2023, 2026). It would appear that some improvement occurs over repeated play; indeed, Degen and Franke (2012), Mayn and Demberg (2023), and Mayn and Demberg (2026) find evidence for small trial-over-trial growth in target selection specific to critical conditions, although this effect is not universal (Franke & Degen, 2016; Sikos et al., 2021b).

### Individual Differences in Multi-Round Games

The picture that emerges above is one where comprehenders, taking part in reference games out of the blue, do not show a particularly robust tendency towards the more sophisticated Gricean reasoning that would be predicted by second-order RSA models or their near cousins. A second strand of research has highlighted that even where evidence suggests sophisticated Gricean reasoning, individual players showed notable variation in these patterns.

The question of individual-level behavior in multi-round reference games was first taken up by Franke and Degen (2016). In one experiment, they analyze data from 51 participants playing as comprehenders across 66 trials, including 12 simple and 12 complex trials as defined above, created using less abstract icons for possible referents (one of three figure types with one of three accessories), and a limited message bank. Average performance in their sample resembled previous studies, with targets selected more often in “simple” trials (77%) than “complex” (57%), although in this case the latter was still found to be significantly above chance. But in fact, individual participants were fairly internally-consistent in each condition: either they seemed to distribute choices at chance between targets and competitors, or they preferred targets in at least 75% of trials. Zooming out, three concentrations emerge: those who were at chance in both conditions, those who selected the target consistently in the “simple” trials but were at chance on “complex” ones, and those who selected targets consistently in both. Franke and Degen fit a hierarchical, heterogenous Bayesian model to this data, explaining individual performance as emerging from either a literal, first-order, or second-order RSA comprehender; the model provides a better fit than a homogeneous model which assumes that all participants are second-order RSA comprehenders, and estimates that individuals sorted into these three classes at roughly 30%, 56%, and 14% probabilities, respectively.

**Figure 2.**
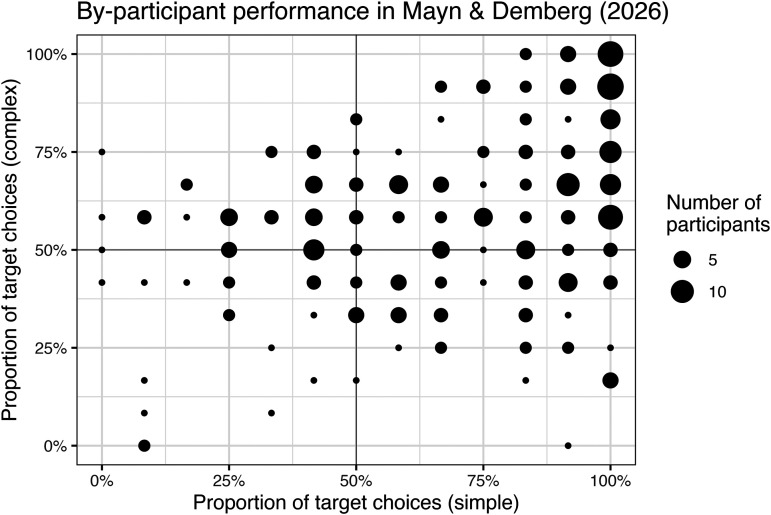
Distribution of by-condition accuracies in Mayn and Demberg (2026).

Mayn and Demberg (2026) followed up on these findings. Collecting an expanded sample of 254 participants, and switching to geometric shape and color stimuli to avoid certain biases in the Franke and Degen (2016) stimuli (see Mayn & Demberg, 2023), the authors nevertheless replicated several aspects of the original results. Performance on simple trials was better than on complex trials (72.1% vs. 62.1% target selections), with the latter again significantly above chance. While individual variability was much less categorical than in Franke and Degen (2016), when Mayn and Demberg (2026) clustered their participants based on performance, three groups again emerged which roughly correspond to the predictions of the three orders of reasoning in the RSA approach (Figure 2). This clustering seems to reveal more literal and second-order comprehenders and fewer first-order comprehenders than observed by Franke and Degen (2016), with the three classes at approximately 34%, 26% and 40%, respectively.^3^

In a subset of 167 participants from their main sample, Mayn and Demberg (2026) also identified cognitive traits which predicted this individual variability, using a battery of individual difference measures. One apparent source of variation came from a construct for Theory of Mind, the ability to take others’ mental states into account and imagine that others’ mental states are different from one’s own. On the proposal that pragmatic inferences require consideration of the expected behavior of a conversational partner, variation in this construct is a very plausible source for individual variation in inferencing. Indeed, assessment of the reference game sample revealed that higher rates of target selection on both simple and complex trials were associated with higher performance on two measures proposed to index Theory of Mind, the Short Story Task (Dodell-Feder et al., 2013) and the Reading the Mind in the Eyes Task (Baron-Cohen et al., 2001). This matches studies on other pragmatic inferences, including scalar implicatures and indirect requests (Fairchild & Papafragou, 2021; Trott & Bergen, 2019, 2020), where a Theory of Mind construct has been linked to higher inference rates.

Mayn and Demberg (2026) also assessed whether differences might also arise from capacities which are not directly implicated in theories of Gricean reasoning, including (a) working memory capacity, measured by the Operation Span task (Unsworth et al., 2005) and the Digit Span Backwards task (Hilbert et al., 2014), and (b) reasoning ability, measured by Raven’s Matrices (Raven & Raven, 2003) and the Cognitive Reflection Test (Frederick, 2005). Previous work by Ryzhova, Mayn and Demberg (2023) provided motivation for the latter construct, having observed that scores on Raven’s Matrices were associated with individual differences in constructing another type of ad-hoc pragmatic meaning, atypicality inferences. Mayn and Demberg (2026) found the same for reference game performance: target selection in simple and complex trials was associated with the reasoning ability construct, in addition to the variation explained by the Theory of Mind construct. (They did not find evidence for any effect of working memory.) The effect of reasoning interacted with trial type, driving the largest increases in accuracy for the simple trials compared to complex ones. The presence of such effects suggests that differences in this task go beyond the resources that a Gricean model of pragmatic competence directly implicates.

### The Outlook for Gricean Reasoning

In all, available evidence suggests that although quantitative models of Gricean inference predict some aspects of human performance in reference games, second-order comprehension in particular is not a typical behavior, and is certainly not a strategy participants will deploy as a default when probed in a single-trial experiment. Even when participants have time to build experience with the reference game format, they vary in their performance, with the patterns expected for second-order comprehension exhibited by only around 40% of participants, at the highest estimate. Indeed, Mayn and Demberg (2026) observe that more than a third of participants respond as if they interpret messages purely literally, without pragmatic inference altogether. As Sikos et al. (2021a) argue, this kind of evidence suggests that second-order cooperative reasoning is not a typical or frequent choice by comprehenders in every given communication scenario. Instead, comprehension behavior in reference game experiments is often better predicted by models which are less sophisticated in their consideration of the speaker.

How does this generalization relate to the general Gricean hypothesis that natural language inferences emerge from a general ability to recognize the goals behind others’ behavior? The conclusions we might draw here hinge on our linking assumptions, the connections we believe to hold between that general ability and participants’ performance in a reference game. If we imagine that this connection is more-or-less direct, things look rather dire for the Gricean hypothesis. That is, if we assume that this general Gricean competence would emerge comparably in reference game performance and natural language inference, we must conclude that this competence is rather poor, and natural-language pragmatic inference must therefore be supported by some other mechanism.

But alternative assumptions are possible if we consider looser connections. Intuitively, there are any number of reasons why a participant may be capable of second-order reasoning, but not demonstrate it robustly in a particular task. Sikos et al. (2021a) offer one possibility, hinging on theories of resource-efficient task execution (Gray et al., 2006; Howes et al., 2009; Krueger et al., 2024; Lieder & Griffiths, 2020). There is no guarantee, Sikos and colleagues note, that a comprehender capable of careful reasoning about the behavior of their partner will see fit to deploy that reasoning in a given experimental task. Under this approach, effortful interpretational procedures should only be expected where a comprehender believes that they will be worthwhile. Perhaps in some cases of real-world language use, this is the case. In other scenarios, like reference games, more efficient approximations of partner behavior might be a rational alternative, like the prior-weighted literal comprehender Sikos and colleagues consider. Hawkins et al. (2021) use a similar resource-rational approach to explain patterns of incomplete perspective-taking in asymmetric-occlusion reference games, where both participants know that some objects visible to the comprehender are hidden from the speaker. Comprehenders, especially in early rounds, sometimes fail to use the speaker’s knowledge limitations to help disambiguate a referring expression; Hawkins et al. (2021) suggest that this is a resource-rational starting point from which participants then adjust when they realize more effort will be necessary to understand a given speaker.

To see whether this hypothesis is indeed a reasonable approach to reference game behavior, we would want to better understand exactly how these sorts of efficiency considerations emerge as participants engage in cooperative reasoning, and how this compares to expected behavior under this hypothesis. We should also consider whether this approach can help explain patterns of individual variation in this task, particularly the correlation with general problem-solving performance. Below, we will take this up in detail, considering how a model of individually-parameterized, resource-rational task adaptation would arbitrate between approximations of partner behavior at different levels of complexity. Ultimately, the model adequately captures several previously-observed patterns in reference game performance, and generates new predictions which we validate in a new experiment. As such, we conclude that such an approach is very plausible.

The parameters we adopt for adaptation come from a recent body of work on the way individuals differ in their response to success and failure in a task, including in tasks like Raven’s Matrices. Basing our approach in this literature provides two benefits: a somewhat generalizable model of factors which influence task adaptation, and in particular, a possible explanation for the observed correlation between Raven’s scores and reference game performance. Before we present our model and test its predictions, in the following section we provide background on this work, and how we envision that it may generalize to the task we are studying here.

## SOURCES OF VARIATION IN RESOURCE-RATIONAL TASK ADAPTATION

For a cognitive agent to engage in resource-rational adaptation for task performance, tuning strategies in search of an optimal trade-off between effort and success (Gray et al., 2006; Krueger et al., 2024), they must have some way of tracking the effort and success associated with previous task execution. This history must then inform the strategies taken in future task execution. In research which considers how this could be accomplished in the human mind in real-time, the predominant approach relies on algorithms for reinforcement learning (Frank et al., 2004; Stocco, 2018; Sutton & Barto, 2018), which could be applied to continuously estimate a utility for each candidate action based on past observations, where relative utility could then determine action selection. For instance, Fu and Anderson (2006) show that a variant of the temporal-difference algorithm of reinforcement learning, ^4^ which prefers choices which lead to rewards with the shortest delay, is enough to explain a suite of action selection effects in human and animal behavior, including incremental tuning of trade-offs between effort and success (see also Stocco, 2018; Stocco et al., 2017). These accounts wield some neuroscientific plausibility, supported by the claim that utility estimation via reward back-propagation is a core function of dopamine pathways originating in the basal ganglia (Barto, 1994; Frank et al., 2004, 2007) with corresponding ERP components associated with changes in future actions (Cohen & Ranganath, 2007; Frank et al., 2005; Holroyd & Coles, 2002).

Within models of human utility estimation via reinforcement learning, one strand of work by Michael J. Frank and colleagues (Frank et al., 2004, 2005, 2007) has argued for the existence of individualized parameters mediating between observed success or failure and changes to estimated utility. Frank’s work considers that successes and failures affect utility estimations via different dopamine pathways, an excitatory pathway which encourages repetition of successful actions, and an inhibitory pathway which discourages repetition of actions which were not successful. Individuals differ, they suggest, in the strength of each of these pathways: the strength of positive feedback *F*_pos_ and the strength of negative feedback *F*_NEG_. In the Probabilistic Stimulus Selection task, a measure of statistical learning introduced by Frank et al. (2004), *F*_pos_ and *F*_NEG_ can be measured independently, by examining behavior of participants trained on binary choice trials with high asymmetries in reward likelihood. A stimulus *A* is very often associated with a reward, while a stimulus *B* is very rarely associated with a reward. *F*_pos_ is evaluated by examining how often participants choose *A* in future trials paired with non-*B* alternatives, i.e., how much they learned a positive value for *A*. *F*_NEG_ is evaluated by examining how often participants avoid *B* in future trials paired with non-*A* alternatives, i.e., how much they learned a negative value for *B*. Both constructs show substantial variation in healthy adult participants (Frank et al., 2005, 2007).

Such individual differences in feedback strength within reinforcement learning networks should affect performance in any task which depends on action selection mechanisms. Stocco et al. (2021) offer a particular example in a model of individual differences in Raven’s Matrices, using the ACT-R framework. Raven’s Matrices problems involve a partial matrix of complex geometric figures, where the participant must recognize and complete patterns in order to identify the nature of a missing cell (Carpenter et al., 1990; Raven et al., 1998). In the model that Stocco and colleagues propose for the task, participants hypothesize potential rules governing the patterns in a subset of the matrix, use the rest of the matrix to confirm that these rules explain the observable feature distribution, and use those rules to reconstruct the missing cell, before finally locating that response among the possible answers. In order to do this efficiently, the agent must quickly identify candidate hypotheses about the distribution of each feature, and quickly penalize them when they prove to be incompatible with the complete matrix. Stocco and colleagues suggest that this hypothesis selection and rejection relies on action selection mechanisms supported by reinforcement learning, and participants require strong *F*_NEG_ to disengage from hypotheses which have recently failed.^5^ Evidence supports this hypothesized connection between variation in learning behavior and variation in Raven’s performance: in a subsequent sample of participants, the authors verify that higher Raven’s Matrices scores are associated with with larger *F*_NEG_ as measured by the Probabilistic Stimulus Selection task of Frank et al. (2004), and in an fMRI follow-up, they find that higher Raven’s scores are also associated with lower BOLD responses in the basal ganglia, which they associate with stronger negative feedback.

We note that the observed link between *F*_NEG_ and Raven’s performance may be driven by more than just disengagement from failed hypotheses. Other research has noted that in free completion of Raven’s Matrices, some participants adopt alternative “eliminative” strategies for solving the problems, rather than the more efficient and effective “generative” strategy outlined above, and this choice controls a large portion of the variance in final scores (Gonthier et al., 2024; Gonthier & Thomassin, 2015; Hayes et al., 2011; Jarosz & Wiley, 2012; Vigneau et al., 2006). Effective selection of global strategy is plausibly also dependent on individualized reinforcement learning mechanisms.

The model in Stocco et al. (2021) also highlights another parameter which may control substantial variation, particular to tasks like Raven’s, where a learner can self-supervise, opting out of continued effort based on available information about the environment. In these cases, learners must, on every attempt at the task, arbitrate between further exploration and execution of a final response which may be sub-optimal, like a guess. Although there are more sophisticated ways that this decision might be modeled, one simple approach is to provide a temporal threshold, at which point if no better response has been identified, the agent makes a guess. We might think of this threshold as a measure of “persistence”, or one’s relative intrinsic value of accuracy over speed. Stocco and colleagues vary this persistence value in their model and they find that it also modulates expected success in Raven’s. While they don’t validate this prediction, it accords with a pattern observed by Thissen (1983) and Cheyette and Piantadosi (2024), that participants who spend more time on more difficult problems tend to score higher on the task. Remarkably, Cheyette and Piantadosi (2024) found that this measure of persistence via response time distribution explained 42% of all task variance in their sample; once this was partialed out, a second construct for task ability captured as little as 3% of task variance.

We use the general approach in the Stocco et al. (2021) model as a starting point for modeling resource-rational strategy selection in our task, including individually-varying parameters for negative feedback strength and persistence. Given the results in Stocco et al. (2021) and Cheyette and Piantadosi (2024), it is possible that the Raven’s Matrices correlation observed in Mayn and Demberg (2026) reflects variation in these constructs, which we might expect to be relevant wherever a task requires motivated exploration over several possible solutions.

## AN ADAPTIVE MODEL OF REFERENCE GAME PERFORMANCE

To formalize how performance in a reference game could depend on resource-rational strategy adaptation, and individual variation therein, we follow Stocco et al. (2021) in constructing a computational model using the ACT-R framework for cognitive modeling (Anderson et al., 2004; Anderson & Lebiere, 1998). In ACT-R, the moment-by-moment trajectory of information processing during cognition is simulated through the selection and execution of production rules, conditional operations which govern how a simulated agent collects, stores, and re-accesses information about the environment as it completes a task. The low-level behavior of our model is controlled by a collection of production rules encoding the assumed patterns of visual attention and information integration necessary to arrive at an interpretation of a message in the reference game, at various levels of pragmatic sophistication. The time-course of that interpretation is determined at a fine level by ACT-R’s modules for eye-movement control, motor control, and memory retrieval. These aspects of behavioral modeling are appealing for our goals—not because we seek or require a careful model of e.g., eye movements in this task, but because with a set of reasonable assumptions, ACT-R is an accessible way to extract predictions of the relative duration and complexity of various reasoning strategies. This then allows us to examine how resource-rational agents would choose between them.

We use pyactr here, a recent Python implementation described in detail in Brasoveanu and Dotlačil (2020). Model code is available in our Supplementary Materials.

While ACT-R has been used for computational modeling across many aspects of language production and comprehension (Brasoveanu & Dotlačil, 2020; Cole & Reitter, 2019; Dotlačil, 2021; Hendriks, 2016; Lewis & Vasishth, 2005; Nicenboim et al., 2016; Patil & Lago, 2021; Reitter et al., 2011) these models have usually capitalized on the framework's quantitative predictions for the speed of cue-based memory retrieval, subject to spreading activation and cue overload. Our model includes memory retrieval, but for us this is largely incidental; instead, we adopt ACT-R here mainly to generate quantitative predictions about strategy selection and learning from experience, following in another line of ACT-R models which have largely addressed phenomena outside of language (Arslan et al., 2017; Fu & Anderson, 2006; Stocco, 2018; Stocco et al., 2017; Taatgen, 2002, although cf. a few language-related models in e.g., Brasoveanu & Dotlačil, 2021; Ceballos et al., 2020; Taatgen & Anderson, 2002; Yang et al., 2021). As introduced in Fu and Anderson (2006), ACT-R estimates the utility of each production rule available within a given model by way of reinforcement learning (Sutton & Barto, 2018), specifically implementing a variant of temporal difference learning, distributing rewards back in time along the multi-step chain of previous productions, weighted by real-time recency of execution. Whenever the model state satisfies the conditions of multiple rules, only the rule with the highest utility (after application of logistic noise) is executed. As in the Stocco et al. (2021) model of Raven’s Matrices, we use this aspect of ACT-R to govern the consideration and rejection of possible solutions, as various model-internal error signals propagate backward to the production rules that generate each solution.

In the rest of this section, we will describe how our model carries out the three main interpretive strategies we define, and how exactly utility learning occurs within and across trials to drive adaptation.

Before moving forward, we will note that the approach taken here allows us to be a bit more precise about the linkage between reasoning and resource-rational strategy selection than previous work in this spirit. As mentioned above, Hawkins et al. (2021) present a Bayesian model capable of resource-rational adaptation to help explain a similar set of empirical phenomena, where the actual performance of participants in a pragmatic task appeared sub-standard compared to the expectations of abstractly rational competence models. As we do, the authors suggest that this could be due to a preference to avoid unnecessary effort, and they show that the tuning of a parameter in their model could approximate how a participant might gradually adjust effort upward to achieve success in an atypical scenario. Nevertheless, they note that their model stops short of modeling a particular “process-level algorithm”, and thus can only treat differences in effort between strategies at an abstract level. This also means their predictions for trial-by-trial adaptation must remain similarly abstract. In contrast, in the present work, we find it useful to adopt a specific, mechanistic approach to resource-rational adaptation, so that we can derive domain-general individual differences in the application of that mechanism. This in turn made it necessary for us to adopt more concrete assumptions about the nature of effort. While the particular assumptions are sure to be incorrect in some way, we think they are a good place to begin. And with them in place, the model allows us to explore realistic consequences of the core hypothesis of resource-rationality, in detail.

### Defining Interpretive Strategies

We equip the model with strategies that correspond roughly to literal, first-order, and second-order reasoning as discussed above. In order to implement these strategies, we do not specify a process of probabilistic reasoning over alternatives, *per se*. Instead, the model considers each possible referent in turn, left to right, ^6^ and makes a binary decision, whether, under the given interpretive strategy, that referent would be maximally probable as the speaker’s intended referent, or not.^7^ Each strategy corresponds to a different amount of visual information which the model will access and evaluate as it makes this decision, see Table 1. Decisions for each referent are stored until all referents have been considered; at that point, if only one referent was maximally probable, the strategy was successful at identifying a response, and that referent is selected. If no referents, or more than one referent satisfied this criterion, the model will attempt to apply another strategy, as will be described in the next section.

**Table 1.** Interpretation strategies defined in the ACT-R model.

	**Goal**	***S*(*M*, *R*) if and only if …**
(A)	Find matches	match(*M*, *R*)
(B)	Find matches w/o other matching messages	match(*M*, *R*) ∧ ∀*M*′[*M*′ ≠ *M* → ¬match(*M*′, *R*)]
(C)	Find matches w/o other unambiguous messages	match(*M*, *R*) ∧ ∀*M*′[(*M*′ ≠ *M* ∧ match(*M*′, *R*)) → ∃*R*′[match(*M*′, *R*′)]]

*S*(*M*, *R*) passes if and only if message *M* can refer to referent *R* under strategy *S*. match(*M*, *R*) passes if and only if message *M* literally matches a feature of referent *R*.

Strategy (A), corresponding to literal interpretation, accepts any referent which matches the feature in the observed message. Evaluation requires only comparison between the referent *R* and a representation of the message *M* held in the model’s focus of attention (the imaginal buffer).

Strategy (B), corresponding to first-order pragmatic interpretation, accepts only referents which match the feature in the observed message and have no other available messages. A literal speaker would be most likely to use that message for such referents. Given our two-feature set-up, these are referents whose other matching message, besides the one which was observed, is not present among the bank of available messages. Evaluation requires, for each referent *R* considered which matches the stored message *M*, an iteration through the available messages *M*′ (visually or in memory^8^), rejecting the referent *R* as soon as another matching message is found, otherwise accepting it once all messages *M*′ have been scanned.

Strategy (C), corresponding to second-order interpretation, accepts only referents which match the feature in the observed message and have no other available messages which would be unambiguous—that is, no other messages that would match only that referent. A first-order speaker would be most likely to use that message for such referents. Given our two-feature set-up, these are referents whose other matching message, besides the one which was observed, is either absent, or else matches another possible referent. Evaluation requires, for each referent *R* considered which matches the stored message, an iteration through the available messages *M*′, as above, and, for each *M*′ which matches referent *R*, an iteration over the alternative referents *R*′, eventually accepting the referent *R* unless a matching *M*′ does not match any alternative referents *R*′.

Like the RSA models which they correspond to, these strategies differ in their expected success across the three conditions we care about, unambiguous controls, simple trials, and complex trials, as summarized in Table 2. Strategy (A) only identifies a unique referent in the controls, and otherwise must make a random guess between potential referents which match the observed message. Strategy (B) can also identify a unique referent in simple trials, but it is only strategy (C) which delivers a unique referent in all types of trial.^9^

**Table 2.** Solving ability of the four strategies.

	**Solving ability**			**RSA equivalent^a^**
	**Control**	**Simple**	**Complex**	
(A)	✓	–	–	literal listener ℒ_0_
(B)	✓	✓	–	first-order listener ℒ_1_
(C)	✓	✓	✓	second-order listener ℒ_2_

^a^ Minimum RSA model capable of solving the same problems.

**Key:** ✓ Strategy identifies correct solution.

–  Strategy cannot find a solution.

In our model, the execution times of each strategy also vary, dependent on noisy execution times for memory retrieval and eye movements, and the order of available referents and messages, but on average, strategy (C) takes about 4.0 s from initiation to correct keypress, and strategy (B) takes about 3.5 s, while strategy (A) is substantially faster at about 1.0 s.

### Iteration and Adaptation

**Figure 3.**
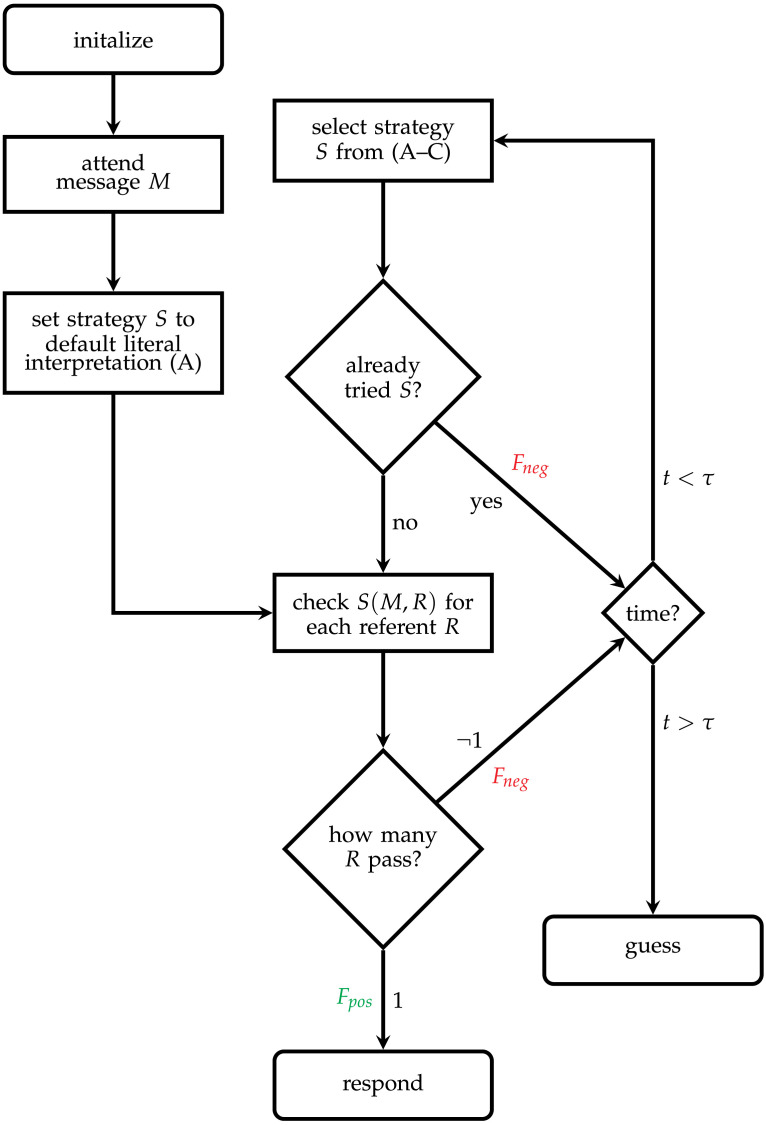
A schematic representation of our model. Refer to Table 1 for relevant definitions.

These three strategies are embedded within a strategy selection, implementation, and disengagement cycle that we adapt from Stocco et al. (2021). We present a simplified schema in Figure 3.

On a first pass, after scanning and storing the observed message, the model will attempt the literal strategy (A), quickly scanning over each possible referent to determine how many literally match. We implement this as a default not because we think it should be maintained as the highest-utility of the three options, but because we think it is probable that regardless of utility, participants applying all strategies complete this as an initial check, especially in our version of the task, where the relevant features are simple, and visual match is easily apprehended.

Whenever a strategy delivers a single optimal referent, as described above, a motor command will be launched to select it, and the model will receive a positive reward, raising the estimated utility for all preceding productions on that cycle, including, on non-initial rounds, the production which initialized the relevant strategy. Otherwise, those productions will be penalized by a negative reward, and the model will move to select an alternative strategy, based on expected utility.

As in the Stocco et al. (2021) model of Raven’s Matrices, fixation on a strategy which has already been attempted for a given problem is possible, when disengagement is not successful—that is, when the utility of the old strategy is still higher than the alternatives after penalization. The first productions fired after initial strategy selection search memory for whether the selected strategy has previously been implemented, and if so, attempt disengagement again. If disengagement is poor, this cycle may require several repetitions before another strategy is successfully identified. While repeated sampling may seem to be an oversimplification of difficulty in identifying alternative strategies, we find it compatible with a more general hypothesis, that successful strategy exploration requires more robust disengagement when there seem to be no plausibly better alternatives. The delay may also correspond to other procedures which we have not modeled here, including trial and error in assembling new strategies.

When unattempted strategies are successfully selected, they are implemented and evaluated in the same way, referent by referent. The cycle of rejection and re-sampling repeats until either some strategy succeeds, or until the model’s internal representation of time spent on the problem (Taatgen et al., 2007) exceeds the model’s persistence threshold. At this point, the model returns a guess; if the last-implemented strategy found two equally-probable referents, the model will respond one of those two at random; otherwise, it will respond with any literally-matching referent. Notice that non-matching referents will never be returned under any circumstance.

Following Stocco et al. (2021), we allow the strength of negative reward *F*_NEG_ and persistence to vary, as individualized parameters. In the simulation to follow, we explore how model predictions vary across possible values for these parameters, and how well this model can explain the variation we see in Mayn and Demberg (2026). In our later experiment, we test the novel predictions of the model in a study with human participants.

## SIMULATION: EVALUATING MODEL PERFORMANCE

In this section, we examine the predictions of our model for how reference game performance will be influenced by individual variation in reinforcement learning. In particular, we report three analyses of model performance: (1) a survey of model predictions under various combinations of *F*_NEG_ and persistence, to demonstrate how they affect the performance of simulated participants; (2) a simulation of Mayn and Demberg (2026), to consider whether this model could reproduce their patterns given a sample from a variable population; (3) a comparison of model fit, pitting our complete model against a variety of simpler ACT-R and RSA models. Our full ACT-R model fares the best in these comparisons, preferable not only because it can explain the relationship with general problem-solving, but also because it better accounts for the fine degrees of variation between participants.

### Survey of Predicted Behavior

We first conducted a broad investigation of simulated behavior across values of *F*_NEG_ and persistence. Simulated participants had one of twenty *F*_NEG_ values between 0.50 and 10.00, and one of ten persistence values between 24 and 33.^10^ Each parameter combination was sampled 25 times, making for a total of 5000 simulations.

Each simulated participant completed a 66-trial version of the reference game task, based directly on Experiment 4 in Mayn and Demberg (2023), and Mayn and Demberg (2026), including 12 simple trials, 12 complex trials, plus 35 unambiguous fillers and 9 completely ambiguous fillers as a control.

Utility learning was enabled, with the default learning rate of 0.2, and noise with the scale parameter 0.6. Initial utilities for the production rules which initiate strategies (A), (B), and (C) were 5.0, −2.5, and −5.0, respectively. Negative utilities for the latter strategies ensure that (A) must be penalized substantially before the model explores these alternative possibilities.^11^ Initial exploration of model performance found that these parameters, together with the tested ranges of *F*_NEG_ and persistence, produced a range of accuracies in simple and complex trials comparable to those observed in Mayn and Demberg (2026). We do not vary these initial utilities here, to allow us to focus on variation contributed by adaptation parameters alone, but see Duff, Mayn and Demberg (2025) for an initial exploration of how different initial utility settings influence the trajectory of adaptation. Other parameter settings were adopted to allow strategies to execute realistically, with an occasional chance for error of memory operations: activation for chunks corresponding to relevant objects extracted from the display (messages, referents) was subject to base-level learning from frequency of access, as well as spreading activation based on the attribute-value pairs in the focus of attention, all with default parameters.^12^

**Figure 4.**
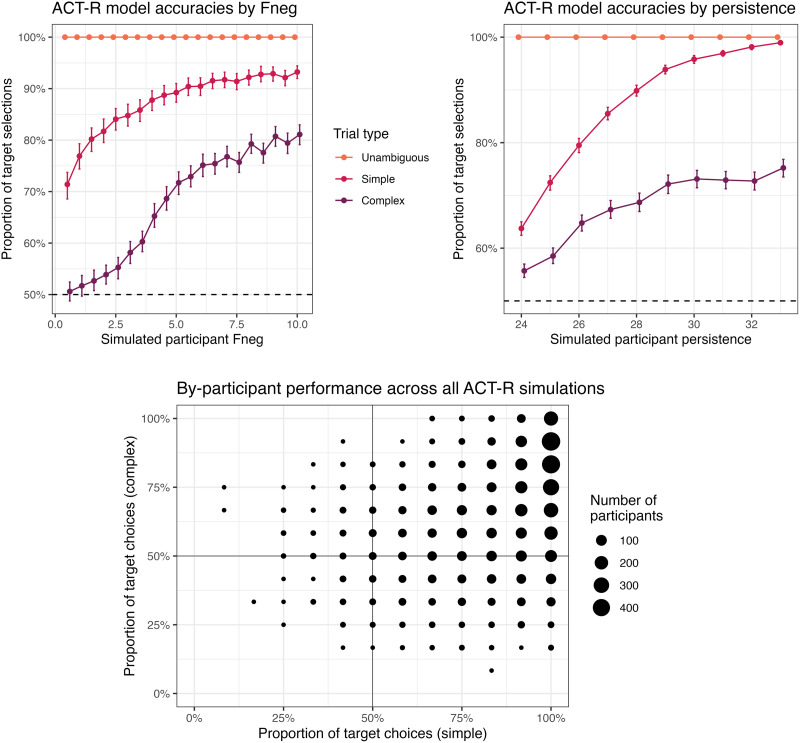
Effects of adaptation parameters on accuracy, by condition, in our general investigation of ACT-R model performance: *F*_NEG_ (top left), and persistence (top right), plus the variety of possible single-participant outcomes across all combinations of these values (bottom).

Results from this broad investigation revealed that expected performance varied widely on the basis of both *F*_NEG_ and persistence, producing overall accuracy ranges comparable to those in Mayn and Demberg (2026), between 50% and 100% in each condition, with more target selections on simple trials than complex ones (Figure 4).

### Simulating Mayn and Demberg (2026)

Having shown that the model can reproduce a range of possible behaviors, we now consider whether our model can recreate the specific patterns of variation in behavior observed in the sample in Mayn and Demberg (2026). We assume that values of *F*_NEG_ and persistence that a participant would exhibit in this task are not uniformly distributed in the population, but concentrated around particular modal values. Therefore, to conduct a proper test of this model against observed data, we want to know whether there is some hypothesized distribution of these two reinforcement learning parameters, such that a random sample from that population fed through our model would demonstrate the observed patterns.

Using the Python package pymc, we fit the mean and standard deviation for independent^13^ normal distributions of *F*_NEG_ and persistence in the populations which would maximize the likelihood of our model generating the Mayn and Demberg (2026) data (Table 3). Expected values are concentrated at the lower end of both scales, but with substantial variation ensuring that higher values are not uncommon.^14^

**Table 3.** Posterior means and 95% highest-density posterior intervals for hyper-parameters governing the distribution of *F*_NEG_ and persistence, fit to Mayn and Demberg (2026) assuming our full ACT-R model.

**Hyper-parameter**	**Mean**	**95% HDPI**
*μ*_*F*_NEG__	1.04	[0.50, 1.78]
*σ*_*F*_NEG__	4.74	[4.08, 5.42]
*μ*_persist_	24.56	[24.00, 25.37]
*σ*_persist_	6.86	[6.05, 7.66]

If our model is an appropriate treatment of the underlying factors here, ACT-R simulations with participants sampled from this distribution should generate data that looks like the patterns in Mayn and Demberg (2026). Indeed, comparing against 228 participants from that experiment, ^15^ a random sample of 228 participants from this distribution reproduces many of the same patterns. To demonstrate these patterns in detail, we examined the parameters of a Bayesian logistic regression fit to one sample of simulated responses. We report the regression in full in Appendix A. In the following subsections, we review the results of this analysis in detail across a few areas of interest, limiting ourselves to those effects and contrasts for which the 95% highest-density posterior intervals excluded 0.

#### Individual Differences.

**Figure 5.**
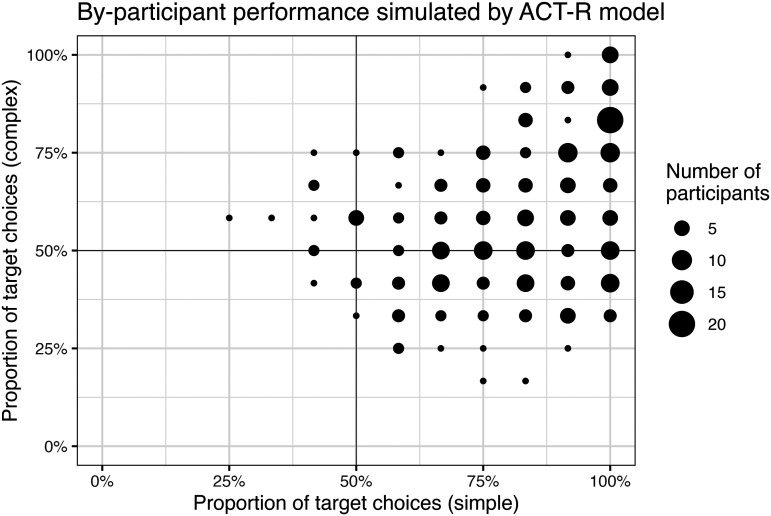
Distribution of by-condition accuracies across agents in our ACT-R simulation of Mayn and Demberg (2026).

Accuracy in the simulated experiment varied across condition, notably better in simple trials (82.23%) vs. complex trials (59.94%). The distribution of individual simulated participants resembles that of Mayn and Demberg (2026) (compare Figure 5 and Figure 2): most participants are above 50% accuracy in simple trials, while most participants are around 50% accuracy in complex trials, except for some of those around 100% accuracy on simple trials. That is, our model succeeds in its principal goal, demonstrating that human-like distributions of individual differences in the reference game could come about from variation in resource-rational strategy selection.

We can probe further into the simulated data to identify particular patterns in the individual differences that this kind of model expects to surface. As expected given general investigation of the model (Figure 4), simulated accuracy in this sample is independently supported by higher *F*_NEG_ and higher persistence. These parameters were associated with smaller gains in accuracy in complex trials than in simple trials, for both *F*_NEG_ and persistence.

**Figure 6.**
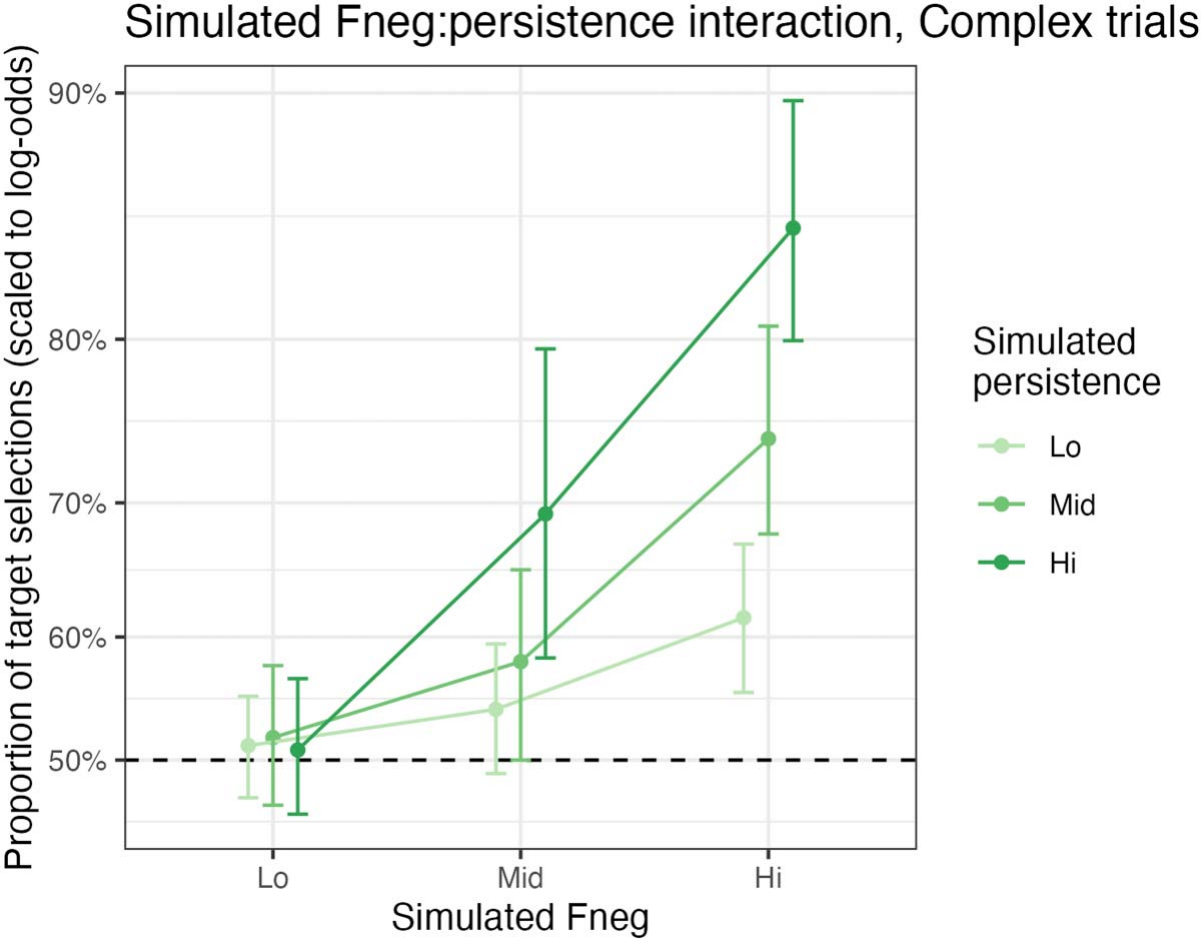
The super-additive interaction between *F*_NEG_ and persistence on accuracy in our ACT-R simulation of Mayn and Demberg (2026), demonstrated on complex trials.

An additional joint interaction reflects a super-additive relationship between the two parameters (Figure 6): *F*_NEG_ has its largest effect (on the log odds scale) at higher values of persistence (and vice versa). In the model, this happens because at higher persistence, more cycles of strategy selection can occur, and a higher *F*_NEG_ will affect each of these rounds.

These aspects of the model’s predictions cannot directly be validated against previous work, as we have no previous insight into the effects of these exact parameters. Still, if we follow the conclusion of Stocco et al. (2021) that the same parameters control variation in problem-solving tasks like Raven’s Matrices, this model would then explain the particular relationship between reference game performance and problem-solving observed in Mayn and Demberg (2026), even mirroring the pattern that individual differences in problem-solving were connected with more substantial changes in performance in simple trials than in complex trials.

#### Adaptation.

**Figure 7.**
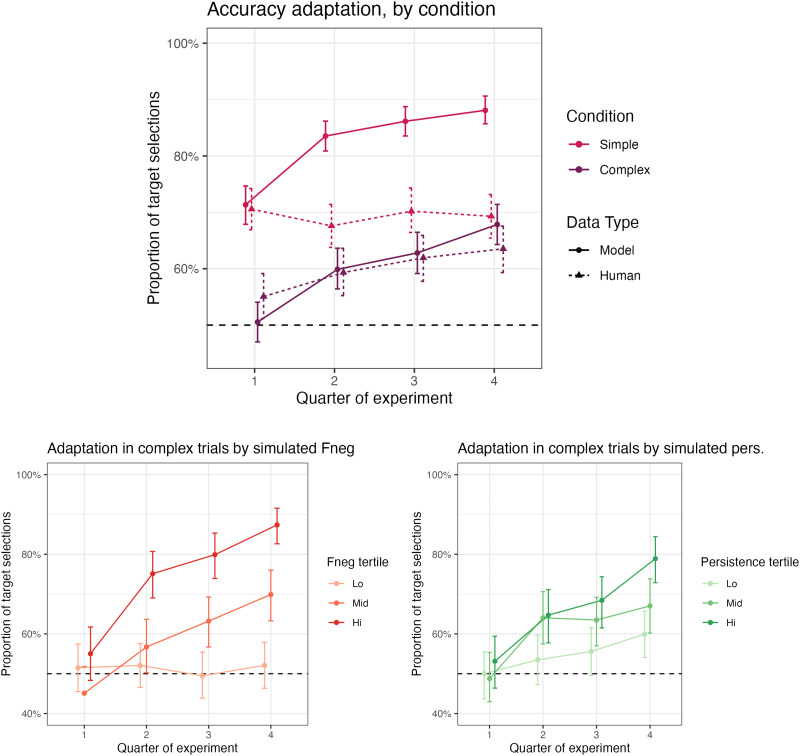
Effects of trial on accuracy, by condition, compared between our ACT-R simulation and the observations in Mayn and Demberg (2026) (top) and then as modulated in our simulation by adaptation parameters (bottom left, bottom right).

Accuracies in the simulation were also subject to improvement with task experience (Figure 7, top). In simple trials, performance improved from an average of 71.35% in the first quarter of the simulation, to 88.10% in the final quarter. In complex trials, performance improved from 50.54% to 67.85%.^16^

This adaptation effect was in turn higher with higher *F*_NEG_ and persistence (Figure 7, bottom). That is, unsurprisingly, we see that in our simulation, these parameters directly affect the rate with which participants adopt more effective interpretation strategies during the task. Indeed, investigation of model traces reveals that larger *F*_NEG_ and persistence are associated with larger increases in the frequency of selecting strategies (B) and (C). While these interactions are an obvious consequences of a model where participant variability only conditions performance differences through differences in adaptation, we note that the connection is only visible to a small degree in a realistic sample.

Looking at the simulated results as a whole, and comparing them to the patterns observed in Mayn and Demberg (2026), expected adaptation rates were closely matched to observed adaptation in complex trials, but on simple trials, expectations are far too high. In fact, in Mayn and Demberg’s study, participants did not improve accuracy in simple trials at all, remaining close to an average of 70% throughout. Why so little change? Within our framework, one explanation would be that participants largely either adapted quickly, to recognize the utility of first-order reasoning within the first few trials (and perhaps eventually learned to prefer second-order reasoning over further exposure), or else they executed literal reasoning throughout. Our model as parameterized here does not easily explain this latter group; even the participants sampled here with the lowest adaptation parameters would be predicted to adapt away from literal interpretation over the course of the experiment. These consistent literal respondents might represent a second, discontinuous subdistribution of participants who engaged in the task at a much lower level than what we have assumed in our model. We will revisit this possibility below, and guard against it in the task design for our own experiment.

#### Response Times.

Our simulation also generates novel predictions about response times; we report these patterns as above, by noting effects supported by a Bayesian log-normal regression (see Appendix A).

First, we observe that correct responses to critical trials arrived much later than those on unambiguous trials. This is intuitive: unambiguous trials should be solved more quickly than trials which require some pragmatic reasoning.

**Figure 8.**
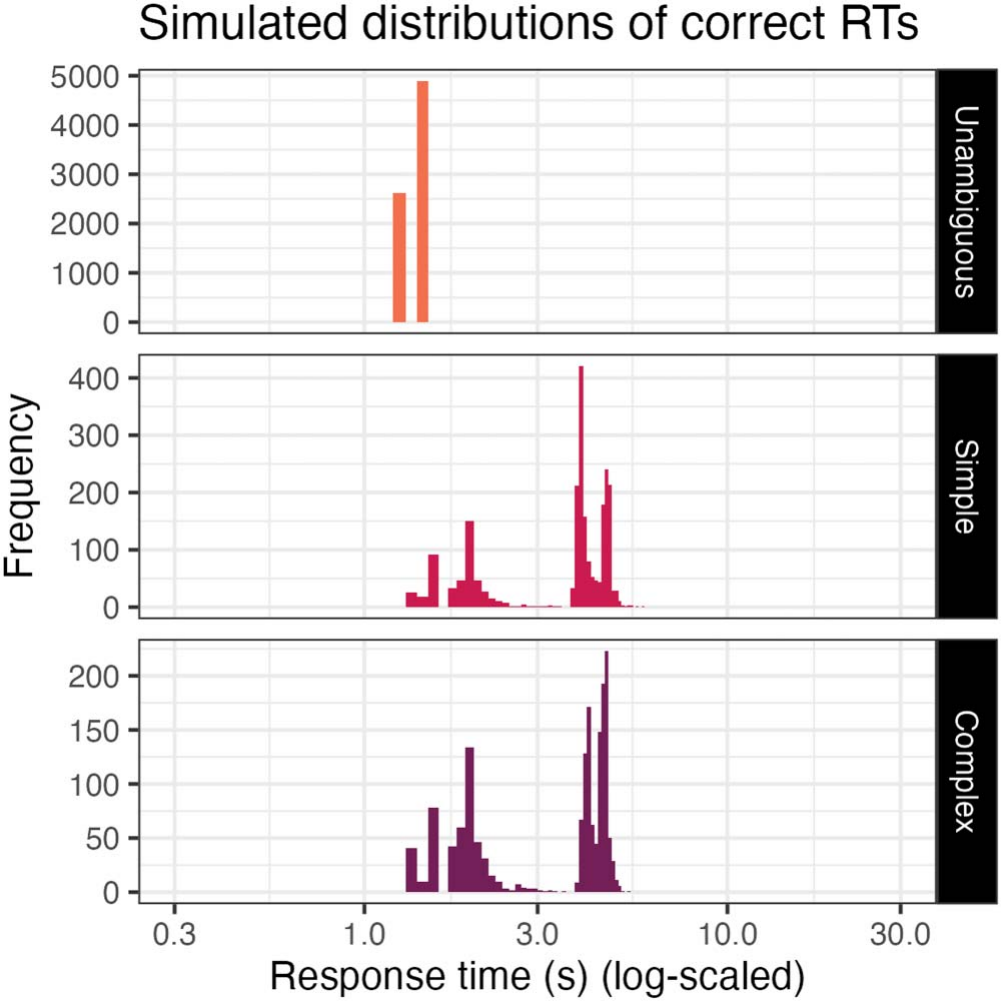
Conditional distributions of response time from our ACT-R simulation.

Other response time predictions are somewhat unintuitive. For instance, comparing within critical trials, we observe that correct responses to complex trials on average arrived earlier than those on simple trials. This may be surprising on its face, as the minimum strategy in our model which can outright solve complex trials, (C), takes longer than the minimum strategy which can solve simple trials, (B). We can understand the pattern better by scrutinizing the distribution of predicted response times in Figure 8. We can see that response time distributions are multimodal in both simple and complex trials; this emerges because there are many ways of obtaining a correct answer, including attempting the trivial strategy (A) before quickly giving up and, by chance, guessing the target correctly.^17^ Because of how much faster this is than executing the other strategies, average response time in a condition is heavily affected by the proportion of correct answers that came from this kind of rapid, lucky guess. And in complex trials, the correct answer was harder to come by through reasoning, so the proportion of correct answers that came from quick guessing was much higher;^18^ it’s this which significantly lowers the average response time.

The same dynamic explains a variety of other response time effects in the simulation. In general, in trials where participants would be more likely to attempt longer strategies (rather than pivoting from (A) straight to a guess), we see longer response times: for instance, in trials of participants with higher *F*_NEG_ and in trials of participants with higher persistence. We take this to be a byproduct of the longer execution times for more effective strategies. Wherever participants more fully preferred more effective strategies, their correct answers would be liable to come from longer processes of reasoning.^19^

### Model Comparison

The above comparisons demonstrated that our model can qualitatively recreate many, although not all, of the patterns found in Mayn and Demberg (2026) as a consequence of sampling from a population where reinforcement learning parameters are variable. Another way of evaluating the quality of our model is to assess whether it outperforms simpler alternatives in the quality of its predictions for unseen data. We compare two classes of alternative model: (A) simplified applications of our ACT-R model, and (B) heterogenous Rational Speech Act models in the spirit of Franke and Degen (2016). More details about model fit and comparison are reported in Appendix B, here we provide a high-level overview.

Among the ACT-R models (A), we include (A1) a fixed-persistence model, *i.e.*, our ACT-R model applied with the assumption that there is a fixed persistence value, and only *F*_NEG_ varies in the population; (A2) a fixed-*F*_NEG_ model, assuming only persistence varies in the population; and (A3), a fully-fixed model, with no meaningful variation in either *F*_NEG_ or persistence. Hyper-parameters for these models were fit independently, following the same procedure as above, estimating the center and (where a parameter was allowed to vary) spread for the two parameters in the population.

Among the RSA models (B), we include (B1) a three-type RSA model, based on Franke and Degen (2016), which assumes that a given participant’s responses come from literal, first-order, or second-order RSA reasoning, distributed with different weights across the population; and (B2) a flexible three-type RSA model, where participants’ likelihoods of being literal, first-order, or second-order comprehenders are allowed to vary across the experiment. To fit these (B) models, we estimated the weights for the three types of comprehension (in B2, these were fit separately for each of the four quarters of the experiment), as well as hyper-parameters *σ*, controlling the amount of noise around the RSA-predicted probability of target response, and *a_α_* and *b_α_*, the shape and scale of a hypothetical Gamma distribution in the population for the ‘greediness’ (*α*) of the probability calculations within the RSA computations (the inverse temperature of the softmax operation).

Predictive accuracy of each model was evaluated by estimating leave-one-out cross-validation metrics (Vehtari et al., 2017) with the arviz package. This procedure estimates how well the model generalizes to held-out data; in our case we see this as a relevant measure for whether a model captures appropriate amounts and patterns of individual variation. Results are presented in Table 4. Our full model outperforms all alternatives we considered, although RSA-based alternatives were competitive with some of the simpler, partially-variable ACT-R alternatives.

**Table 4.** Comparing model fit in terms of expected log predictive density (ELPD) estimates, between our full model and several alternatives.

**Model**	${\hat{\text{ELPD}}}_{LOO}$	**Std. Error**
Our Model	−4183.52	90.00
(A2) ACT-R with Fixed *F*_*neg*_	−4566.29	107.14
(B1) Three-Type RSA	−4568.20	33.96
(B2) Flexible Three-Type RSA	−4569.39	33.93
(A1) ACT-R with Fixed Pers.	–4635.86	54.47
(A3) Fully-Fixed ACT-R	−4701.21	44.83

Our full model’s predictive advantage over the simplified ACT-R alternatives (A1–3) suggests that the complexity of assuming multiple sources of individual differences is merited in the data. It may be surprising that our full model also outperforms a more flexible alternative like (B2), the Flexible Three-Type RSA model. The latter is in many ways a close relative of our model, sharing a three-way distinction between reasoning types, but offers more flexibility in that the shifting of rates of higher reasoning types is not being constrained by underlying assumptions about reinforcement learning. On investigation of model posterior predictions, we note that in fact, with its greater flexibility, the latter model struggles to accurately generalize, overestimating variance between participants, and often underestimating overall performance. Perhaps more appropriate parameters for such a model could be fit against a larger dataset; nevertheless, the fact that the restrictions of our full model allow more efficient generalization suggests that these restrictions are well-aligned with observed behavior.

### Discussion

Simulated data from our ACT-R model serve as a proof of concept for the hypothesis we develop above: a population of agents who rely on resource-rational strategy selection as part of reference game performance would exhibit (a) observed ranges of variation in accuracy, and (b) some of the observed adaptation effects over the duration of the experiment. Although alignment is not perfect, comparison with a range of other similar models reveals that the particular assumptions of resource-rational strategy selection are best-suited to predict the patterns we see in previously-collected data.

Our model also makes several predictions regarding the individual parameters governing adaptation behavior, and how they in particular lead to observed variation in accuracy. Increases in two model-internal parameters, the strength of negative feedback (*F*_NEG_) and persistence, lead to faster adaptation. It’s this faster adaptation which leads to greater use of pragmatic interpretive strategies, and therefore higher rates of success.

Our interest in these parameters was motivated by their connection to performance on problem-solving tasks. Because our model predicts a dependence between reinforcement learning parameters and reference game performance, and previous work by Stocco et al. (2021) illustrates a dependence between the same parameters and Raven’s Matrices, we can now offer an explanation for the correlation between individual behavior in the reference game and Raven’s. We contend that both tasks could be supported by these implicit domain-general constructs. Although we have shown that our model could explain this pattern, we don’t yet have any experimental evidence for the claim that these parameters are actually implicated in reference game behavior.

By simulating the time-course of message interpretation, our model also generates a completely new class of predictions regarding response time, an aspect of reference game performance which has remained unexplored in previous work. In our simulations, correct response times were slower on trials that required any pragmatic reasoning, as correct responses on those trials were more likely to come after applying longer, more complicated interpretive strategies. Simulated participants with faster adaptation parameters were expected to have the strongest tendency towards these longer strategies, and as a result showed especially slow response times. From previous work, we do not yet know whether these predictions are met.^20^

In sum, by simulating a mechanistic model of resource-rational adaptation for this task, we now have a proof of concept that this approach can largely produce appropriate variation, and we also have clarity on new predictions. As a general rule, we expect that variation in domain-general parameters which drive adaptation could affect how quickly a participant decides to engage more resources in a pragmatic interpretation task. In particular, our model shows how *F*_NEG_ and/or persistence would be possible candidates for those parameters—if we are on the right track, and if we can estimate these two parameters, one or both should correlate with reference game performance, and should explain part of the shared variation with Raven’s. As a secondary prediction from this model, if we collected response times, we should be able to see evidence of extra cognitive effort being required on more complicated trials. In our experiment below, we collect new data to test these predictions.

## EXPERIMENT: MEASURING THE ROLES OF FNEG AND PERSISTENCE

Our ACT-R model provides a proof of concept that individualized parameters for reinforcement learning would predict reference game performance, if participants were applying general adaptation mechanisms to identify resource-rational strategies for completing the task. We now present the results of a preregistered follow-up which further tests the predictions of our model in a new sample of actual reference game performance, collected in tandem with secondary tasks to estimate participants’ *F*_NEG_ and persistence. The preregistration is accessible on OSF at osf.io/56gnf.

### Participants

We aimed to recruit 150 native English speakers with minimal approval rate of 95% via the crowdsourcing platform Prolific. As participants were recruited, two tasks provided measures used as rapid exclusion criteria, which we describe below. Participants who failed either of these criteria were excluded from analysis, and new participants were recruited in their place. The final sample featured 149 unique participants: late in analysis, we discovered that due to technical error, one participant completed the experiment twice, and their second set of results was removed from subsequent analysis without replacement.

### Materials

#### Reference Game.

In this experiment, we simplify the reference game design used in Franke and Degen (2016), Mayn and Demberg (2023), and Mayn and Demberg (2026), using a 36-trial experiment including 8 simple trials, 8 complex trials, and 20 unambiguous fillers as a control. Of these 20 unambiguous filler trials, in 4 trials, all features were unique across the three objects. A further 8 trials had the same display as the 8 simple trials, except that the target was the competitor (4 trials) or the distractor (4 trials) from the corresponding simple trial. The remaining 8 trials had an identical display to the complex trials, where the target was the competitor from the complex condition. All trials used the shape (circle, triangle, square) and color (red, green, blue) feature space as advocated by Mayn and Demberg (2023). The message bank always included the same four possible messages (circle, triangle, red, green), and the order of possible referents on each trial and the order of trials were randomized for each (simulated or human) participant.

This shorter design was chosen so that it would minimize potential sources of uncontrolled variance in human performance, and allow easier comparison with the behavior of the ACT-R model. One change from previous designs was the number of trials in the simple and complex conditions. With a fixed message bank and two three-valued features, there are only 8 unique combinations of message and possible referents that produce the “simple” pattern, and another 8 which produce the “complex” pattern. Franke and Degen (2016) generated 12 trials in each condition by repeating some combinations with different randomized referent order, but we chose a smaller design to avoid the risk of memory effects in our model or our participants.

We made two further changes from previous designs to help ensure that human participants, like the model, had a strong baseline motivation to interpret messages thoughtfully, and would not assume that guessing was an appropriate strategy except when they had exhausted other options. First, we avoided the use of completely ambiguous fillers, which in previous studies have included multiple possible referents with the exact same features. As a result, for all trials, second-order interpretation yields a uniquely-likely target. Second, we provided participants with feedback after each trial, indicating whether their response was the intended target. We suspected that removing obviously-unsolveable trials and reminding participants that there were correct answers in all trials could promote participants’ willingness to engage fully in the game. This could, among other changes, reduce the chances of observing a large proportion of uniformly-literal participants.

Feedback has the added benefit of helping dissuade participants from a rare interpretative strategy relying on implicit negation (Misyak et al., 2016), selecting the single referent in simple or complex trials which does not match the observed message. Based on participants’ self-reported strategies at the end of the task, Mayn and Demberg (2026) report that about one in twenty participants settled on this approach in the absence of feedback, and were excluded from further analysis. Because the implicit negation strategy wasn’t included in our model, we wanted to ensure that our participants did not adopt it either. (This appeared to be successful, as we observed very few cases of this type of response.)

Participants were told at the outset of the task that they would be trying to identify the referent of messages sent to them by a participant of an earlier study. They then completed a short section of pre-training, where they were asked to try out completing the task that the prior participant had completed (cf. Sikos et al., 2021b), choosing messages from the message bank to help a future participant select a highlighted option from a set of three available referents.

After this brief pre-training, participants moved on to the main body of the reference game task. Participants were instructed to click on the referent that they thought the previous participant had intended. Responses and response times were collected on each trial.^21^

Participants who achieved below 80% accuracy on unambiguous trials were excluded from analysis and replaced.

#### Individual Difference Tasks.

To measure *F*_NEG_, we collected performance on the **Probabilistic Stimulus Selection** task (Frank et al., 2004), following Stocco et al. (2021). On each trial, participants had to select between two stimuli and received feedback on whether their selection was correct. There were six stimuli in total. In the training phase, the six stimuli were always presented in three pairs, where each stimulus in a pair was associated with a certain probability of success (A vs. B = 80%/20%, C vs. D = 70%/30%, E vs. F = 60%/40%). As in Frank et al. (2004), the stimuli were six characters from Japanese writing systems, and their assignment to a condition (i.e., which character corresponded to A, B, etc.) was randomized for each participant.

**Figure 9.**
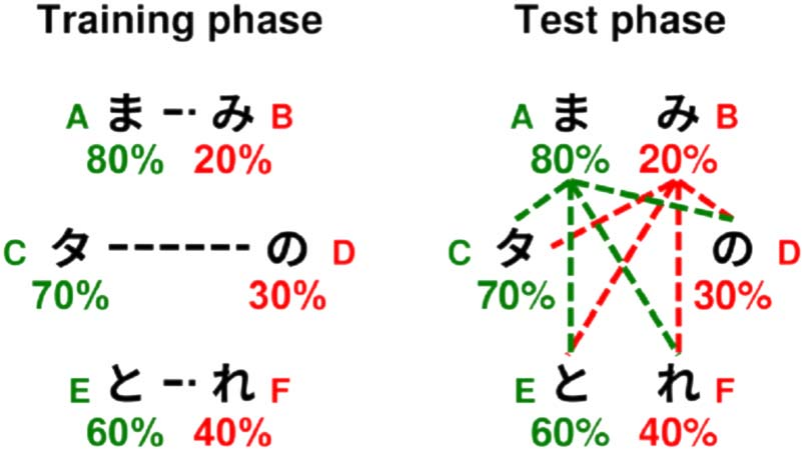
An overview of the training and test phases of the Probabilistic Stimuli Selection Task, from Stocco et al. (2021).

The training phase consisted of 60 trials total, 20 trials for each pair. At the end of the training phase, participants’ selection probabilities were checked to ensure that they had learned some of the asymmetries in reward likelihood. If selection probabilities surpassed preset thresholds (selecting A at least 65% of the time, C at least 60% of the time, and E at least 50% of the time), they progressed to the test phase. Otherwise, the whole training phase was repeated. If a participant did not reach the accuracy threshold after six passes through the training phase, they automatically progressed to the test phase. In the test phase, participants again had to make selections between pairs of characters, but this time they were shown all 15 possible combinations of characters and not just the three they had seen during training. Each of the possible character combinations was presented 4 times, for a total of 60 trials. On the test phase, participants did not receive feedback. Figure 9 shows a visualization of the two phases of the task.

The measure of *F*_NEG_ is then calculated using the proportion of B choices during the test phase in the pairings not presented during training (i.e., B in combination with characters other than A). In our analysis, we reverse this proportion, so that a higher score corresponds to stronger tendency to avoid the least successful outcome.

In our preregistration, we planned to exclude and replace participants who completed the maximum six passes through the training phase without reaching the learning targets. As more than 40% of participants in our sample failed this criterion, we decided that it would not be feasible to apply it.^22^ Although this departed from the preregistered analysis plan, we note that differences in the learning behavior of these participants, including their failure to pass the training criteria, can still plausibly be attributed to the variation in learning which the task is intended to measure. To this point, we observe that previous work has never excluded such participants.

To measure persistence, we constructed a novel **Anagram Persistence** task, inspired by Eisenberger and Leonard (1980) and Ventura and Shute (2013). Participants saw 20 trials with five-letter sequences. They were told that each sequence was an anagram, and had a unique English language solution that participants could type into a textbox. On submission of an attempted solution, the response was checked. Correct responses were accepted, and moved the participant to the next trial, while incorrect responses were rejected, and prompted the participant to try again. There was no limit on the number of attempts permitted. Next to the textbox, there was also a “Skip” button, which allowed participants to skip the trial at any point.

Of the 20 trials, 10 had a solution which is a high-frequency word, 5 had a mid-frequency solution, and 5 had no possible solution. The index of persistence was computed as the ratio of the average time people spent on the impossible trials before skipping, to the average time they spent on correctly answered high-frequency trials. Participants who skipped more than 5 out of 10 simple anagrams were excluded from analysis.

We use this as our main measure of persistence, but because it has not been used in this exact form in previous research, we also included two shorter, previously-validated persistence tasks described below. If the measure we compute from the Anagram Persistence task is a valid measure of generalized persistence, it should be correlated with the persistence measures in these secondary tasks.

The **Short Grit Scale** (Duckworth & Quinn, 2009) was collected as a self-reported index of persistence. It is an eight-question questionnaire, where each question asked participants to indicate agreement or disagreement with a self-description (e.g., “I am self-disciplined”) on a five-point scale. The score is the average of provided response values, after appropriate reversal.

We also collected **Raven’s Matrices** (Raven & Raven, 2003) to verify the relationship with performance on the reference game observed in Mayn and Demberg (2026), as well as its relationship with *F*_NEG_ and persistence, extending Stocco et al. (2021). Since it has been shown that performance on a short nine-question version of Raven’s Matrices correlated almost perfectly with performance on the full version of the task (Bilker et al., 2012), we administered a short 11-question version. Each trial showed a pattern with a missing cell, and participants had to select one of the six to eight answer options. The items were presented in a fixed order of increasing difficulty. In an adjustment made to help provide another secondary persistence measure, the final item had a definable answer which was not among the provided options, and participants had to select one of the available incorrect answers in order to continue, as in Dale et al. (2018). For this task, there was a timer at the top of the screen, which displayed the total amount of time participants had spent on the task up until that point. Participants were told that while their time isn’t limited, they should avoid thinking too long.

Scores for this task were computed as the number of correct answers to the first 10 questions. Response time on the last impossible item (the time participants took before selecting an incorrect answer) was used as an index of persistence. Dale et al. (2018) have previously observed that measures related to this response time are correlated with scores on the Short Grit Scale (and response time on another impossible problem-solving task).

While the focus of the current work is on the relationship between persistence and negative feedback strength and the reference game, we additionally collected the **Short Story Task** (Dodell-Feder et al., 2013) as a Theory of Mind measure, since Mayn and Demberg (2026) had found a main effect of Theory of Mind on reference game performance. In this task, participants read the short story “The End of Everything” by E. Hemingway. They were then asked to briefly summarize the story, after which they answered 13 open-answer questions, 8 of which were questions about characters’ feelings and mental states and 5 were comprehension questions. Participants’ answers were scored 0–2 using the rubric provided by Dodell-Feder et al. (2013).

The relevant score for Theory of Mind is the sum of scores of the mental state questions. Participants who scored less than half of the available points on the comprehension questions were excluded from secondary analyses involving this measure. (Because this criterion required hand-coding, it was applied after data collection, and these participants were not replaced.)

### Procedure

Participants completed the tasks in the following order: reference game, Raven’s Matrices, Probabilistic Stimuli Selection, Anagram Persistence, Short Story Task, Short Grit Scale. The whole experiment took participants about 57 minutes to complete on average.

### Results

We present the results of this experiment below, organized into three main subsections. First, we compare average performance across conditions and how it changed over the duration of the experiment. This gives us the chance to confirm that the sample replicated expected global patterns of reference game behavior, and present a first glimpse of typical response times. Second, we survey our individual difference measures, assuring that they are suitable for testing the key hypotheses of our model, before then assessing our main predictions about the effects of *F*_NEG_ and persistence on reference game behavior. For now, we focus on the properties of the data as they stand—in the following section, we then assess our ACT-R model’s ability to recreate these patterns compare these patterns with hyper-parameters fitted to the new data.

#### Global Patterns in Reference Game Performance.

We first explore whether participants as a whole exhibited patterns of adaptation and conditional differences in accuracy observed in previous studies. As above, we base our discussion on those effects which were supported by a logistic mixed-effects regression fit to response data, and a log-normal mixed-effects regression fit to response time data, see Appendix A for specifications and complete results.

**Figure 10.**
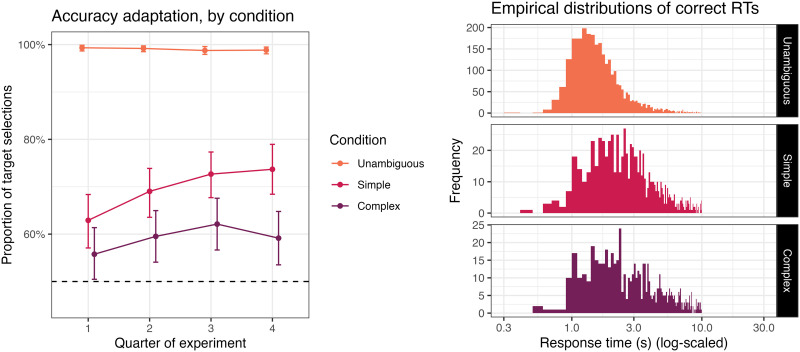
Visualizing global patterns in our human data, including adaptation (left), and response time distributions (right).

Accuracy indeed differed across conditions, and improved over the course of the experiment (Figure 10, left), matching and somewhat exceeding the adaptation observed in previous studies. On average, participants were less accurate on the critical trials than on the unambiguous fillers, and less accurate on the complex trials than on the simple ones, falling from 69.7% to 59.0%. While there was no main effect of trial order, marginal comparisons reveal that accuracy improved with experience specifically on critical trials. In simple trials, we observe an increase in accuracy from 62.9% in the first quarter of the experiment to 73.7% in the final quarter; for complex trials, this was an increase from 55.8% to 59.2%.^23^

Differences in response times between conditions (Figure 10, right) somewhat resembled the patterns of our ACT-R simulation above, where correct responses were slower for the critical trials, but slightly faster for complex than simple trials, owing to a higher proportion of guesses. Here, correct responses were indeed much slower on simple and complex trials, at medians of 3.13 s and 3.67 s, than on the unambiguous fillers, at a median 1.71 s. While complex trials in our human data were the slowest, the contrast between simple and complex trials did not pass our threshold for notability in this analysis.

As is typical in many experiments, participants sped up during the course of the experiment, across all trial types. For instance, correct responses to complex trials reduced from a median of 5.15 s in the first quarter of the experiment to a median of 3.09 s in the final quarter.

In sum, the global results confirm patterns of accuracy observed in previous studies, which we expect to be well-explained by our ACT-R model. At the same time, our first glimpses of response times in this task are partially in line with our model’s general expectations for conditional difficulty.

#### Profiling individual difference measures

**Figure 11.**
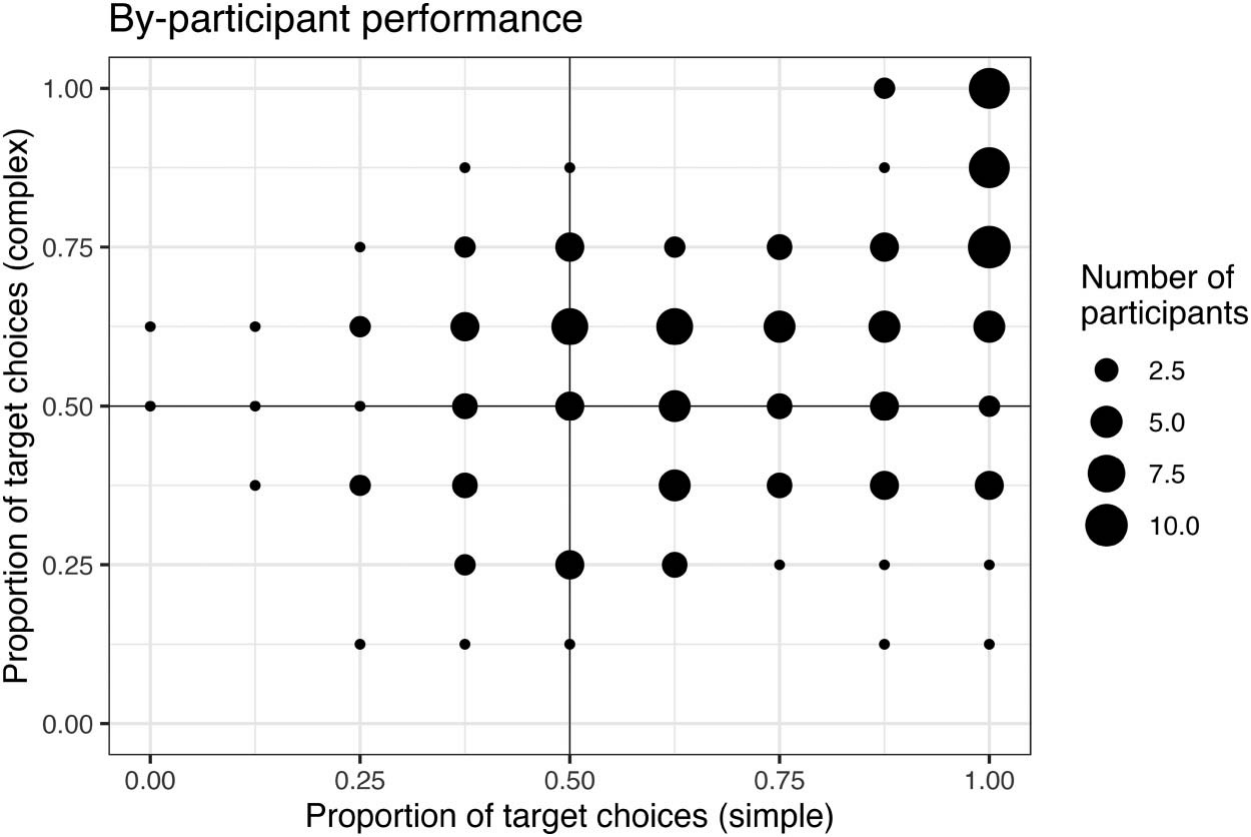
Distribution of by-condition accuracies in our human experiment.

We also succeed in replicating a typical distribution of individual differences in reference game performance (Figure 11). Participants again varied in accuracy in simple and complex trials, largely between 50% and 100%, with complex accuracy most often lower than simple. We are most interested in how this variation relates to variation in the other measures we collected, and in particular whether we can predict this variation in terms of *F*_NEG_ and persistence, as expected by our model.

Before we investigate these particular relationships, first we profile the distributions of these secondary measures in their own right. Descriptive statistics of the individual difference measures are included in Table 5. No measure appears to exhibit ceiling effects.

**Table 5.** Descriptive statistics of the collected individual difference measures.

**Measure**	**Mean**	***SD***	**Observed range**	**Possible range**
PSS *F*_NEG_	0.63	0.25	0.06−1.00	0.00−1.00
Anagram persistence	4.03	2.81	0.67−20.22	0.00–∞
Grit	3.27	0.86	1.13–5.00	1.00–5.00
Raven’s persistence	39.84 s	34.59 s	2.43 s–170.59 s	0.00–∞
Raven’s Matrices	5.39	2.32	0–9	0–10
SST	8.30	3.01	1–15	0–16

As noted above, our main measure of persistence, from the Anagram Persistence Task, has not previously been validated for this construct. We offer a partial cross-validation here by way of comparisons with the two other persistence measures we collected: time spent on an impossible Raven’s problem, and self-responses on the Short Grit Scale. Participants who show higher persistence on the Anagram task also spent significantly longer on the impossible Raven’s problem, *r* = 0.22, *p* = 0.006.^24^ On the other hand, persistence on the Anagram task appears to be inversely correlated with scores on the Grit scale, *r* = –0.19, *p* = 0.019. In fact, this latter correlation is driven by two individuals who have much higher Anagram persistence scores than the rest. If they are excluded, the relationship between Anagram persistence and Raven’s persistence remains strong, but there is no longer any significant relationship between Anagram persistence and the Grit score, *r* = –0.11, *p* = 0.18. There is likewise no significant relationship between Raven’s persistence and the Grit score, *r* = –0.13, *p* = 0.12, in contrast with the findings in Dale et al. (2018). Even though Grit score did not correlate with our Anagram persistence measure, we view this cross-validation as largely successful. We are not particularly concerned about the behavior of the Grit score, because (a) our experimental measures of persistence do correlate with each other, but neither correlates with Grit scores, and (b) weak correlations between self-report and experimental measures of the same construct have been often observed, and it has been argued that distinct processes may underlie the two types of measures (Dang et al., 2020). We go forward using the Anagram persistence score as our metric of persistence.

**Figure 12.**
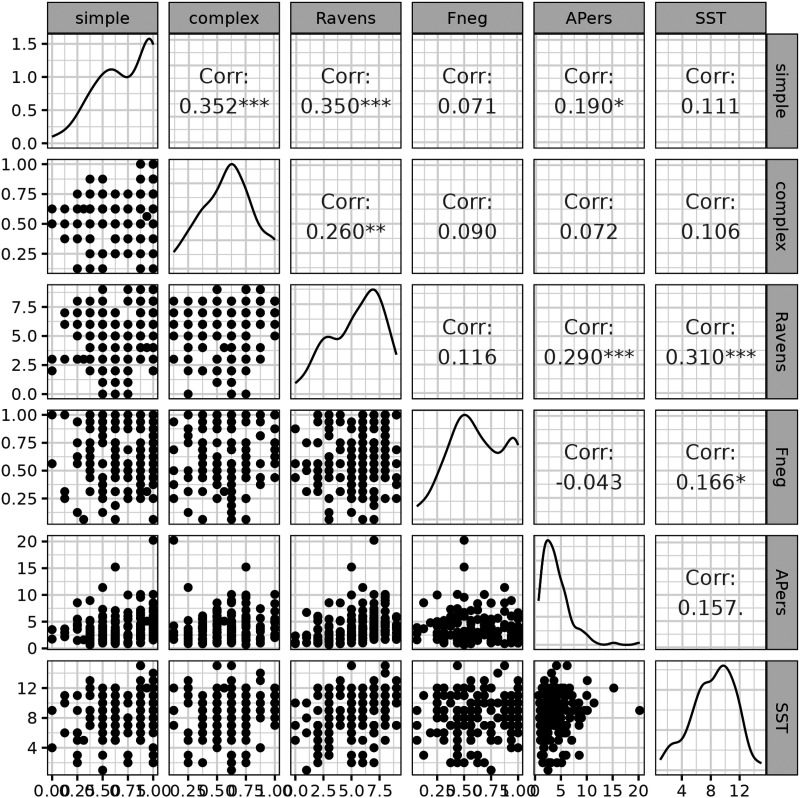
Correlation among individual difference measures.

The correlation matrix of the individual differences is reported in Figure 12. While many measures are weakly to moderately correlated with each other, note that there is no relationship between our two individual difference measures of interest, *F*_NEG_ and persistence, *r* = −0.043, *p* = 0.84, suggesting that the two are independent constructs.

#### Effects of *F*_NEG_ and persistence.

**Figure 13.**
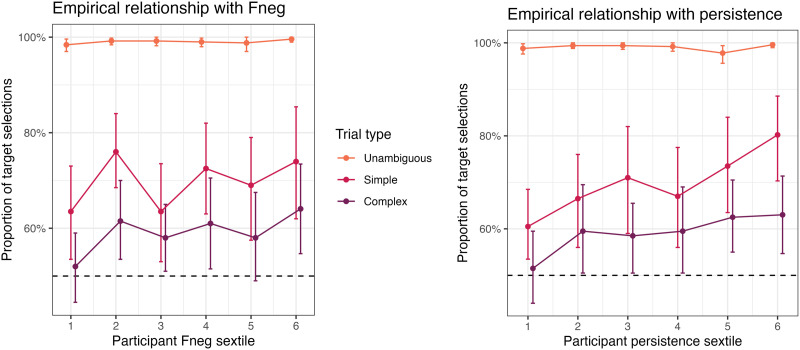
Visualizing marginal individual difference patterns in our human accuracy data for *F*_NEG_ (left), and persistence (right).

We turn now to the relationships between these parameters and participants’ performance on critical trials in the reference game (Figure 13). For complete details of our analysis, see Appendix A. As predicted, we observe that participants with higher persistence scores performed better on critical trials in the reference game, $\hat{\beta }$ = 0.32. There is a smaller positive trend for *F*_NEG_ as well, although the 95% highest-density posterior interval includes 0, $\hat{\beta }$ = 0.17, 95% HDPI = [−0.05, 0.40]. There is also a trend of an interaction of persistence with condition, which suggests that it is associated with smaller accuracy gains in the complex condition than in the simple condition, although the 95% highest-density posterior interval again includes 0, $\hat{\beta }$ = −0.15, 95% HDPI = [−0.32, 0.02].

Despite our simulation’s prediction of slower response times for individuals with stronger adaptation parameters, we observe no notable effects of our individual difference measures on response times here. Nevertheless, our models accounting for individual differences did provide new evidence that participants took longer on complex trials than on simple ones, an effect which was not notable in the corresponding analysis without individual differences.

#### Other Individual Difference Effects.

While our main analyses focus on the role of *F*_NEG_ and persistence, we conducted additional regression analyses where we also added Raven’s and SST as predictors. Here we briefly mention the key results; the full models are included in Appendix C. We first attempted a partial replication of Mayn and Demberg (2026), examining Raven’s and SST as the only individual difference predictors of interest. Consistently with the previous results, we find that participants in our sample with higher Raven’s scores tend to respond more pragmatically on the reference game, and this produces larger improvement on simple trials than on complex trials. We also replicate the Stocco et al. (2021) observation that *F*_NEG_ predicts higher Raven’s scores, and validate for the first time their additional hypothesis that persistence should as well. These lower-level variables therefore can explain some of the shared relationship between Raven’s and the reference game, but they do not explain all of it: in a second analysis, we found that Raven’s score helped predict residual variance which *F*_NEG_ and persistence could not capture on their own. We take this to suggest that there are other general mechanisms at work supporting both tasks, surely of interest for further work.

In contrast to Mayn and Demberg (2026), where a construct composed of Theory of Mind tasks also predicted increased pragmatic behavior on the reference game, here we only observe a trend of a relationship between SST performance and reference game performance. We speculate that this difference in results may have something to do with our changes to the main task. For instance, the feedback participants received after every trial may have reduced the relevance of individual differences in Theory of Mind ability by more heavily motivating all participants to carefully consider speaker intentions, even those demonstrating less Theory of Mind ability in other tasks. Nevertheless, a reviewer points out that Trott and Bergen (2020) in fact observe an opposite effect, where raising the salience of a speaker’s epistemic state in a task also raises the observed relationship between that task and the SST Theory of Mind ability score. We leave further scrutiny of these differences to future work.

### Model Fit and Comparison

Qualitatively, several results here are reminiscent with the patterns extracted from our model in the simulations above—in particular, robust adaptation, and individual variation correlated with reinforcement learning parameters. To further assess how well the model could explain these patterns, we again conduct simulations using hyper-parameters for *F*_NEG_ and persistence fit to the data.

As before, we assembled the expected behavior for participants within the same 200 combinations of *F*_NEG_ and persistence, now on the 36-trial version of the task, and then used Bayesian estimation to fit the center and spread of normal population distributions over both parameters. The resulting values (Table 6) suggest asymmetric variation, coming more from persistence than *F*_NEG_. Higher centers for both distributions would also seem to indicate more robust reinforcement learning in this experiment compared to the analysis of Mayn and Demberg (2026) above, in order to explain the generally higher performance we see here. This upward shift may be due to the changes in the experimental procedure, intended to encourage the assumption that there was a correct answer. It seems reasonable to assume that the *F*_NEG_ and persistence constructs hypothesized for a participant in any given task are not general, static individual traits, but are largely sensitive to the motivation the participant brings to the task.

**Table 6.** Posterior means and 95% highest-density posterior intervals for hyper-parameters governing the distribution of *F*_NEG_ and persistence, fit to our new data assuming our full ACT-R model.

**Hyper-parameter**	**Mean**	**95% HDPI**
*μ_F_NEG__*	9.13	[7.78, 10.49]
*σ_F_NEG__*	4.67	[3.70, 5.60]
*μ*_persist_	27.50	[25.73, 29.35]
*σ*_persist_	6.66	[5.70, 7.62]

We can again simulate model performance by examining samples from the population (see Appendix A). Several aspects of the data are well-reconstructed here by the model. This model can reconstruct adaptation in both conditions, including greater adaptation in simple trials than complex trials. Nicely, in this case, this pattern of adaptation is what we see in the observed human data; compare to the Mayn and Demberg (2026) data, which showed asymmetries in adaptation which our model could not reproduce (Figure 7). Crucially, our model again expects patterns where individual differences in reinforcement learning give rise to different behavior; in this case, we have independent evidence for such a dependency from the relationships between tasks.

A few departures are not surprising or interesting. First, the model cannot reproduce the observed speedup in responses across the experiment, predicting instead that a greater degree of complex reasoning will slow down the model slightly at later points. It is a known pattern that response time in experimental tasks generally speeds up over subsequent repetitions (e.g., von Bastian et al., 2022), and this pattern is easily explained by any number of additional hypotheses which were not reflected in the model. For one, in ACT-R and similar frameworks, this pattern can emerge by automatizing common chains of productions into more efficient, single-execution routines, a process known as production compilation (Taatgen, 2013; Taatgen & Lee, 2003), which we left out of this model for simplicity.^25^ Elsewhere, the model has unmet expectations for effects of *F*_NEG_ and persistence on adaptation speed and RT—at least the former seems like a critical prediction of our approach here, but we note that within a single sample of this size, even our idealized model expects these effects to be rather small. It may be that we just cannot detect such effects in the presence of realistic noise. The same can be said of the predicted super-additive interaction between *F*_NEG_ and persistence. Finally, the model continues to predict that the average RT in simple conditions should be slightly slower than that in complex conditions (as in Figure 8)—we now see that this contrasts with actual observed RTs, where complex conditions are taking longer as would be intuitive. This particular model prediction seems non-critical, as it is driven by the particular mixture of guessing vs. more careful comprehension in each condition; we will revisit this topic below.

**Figure 14.**
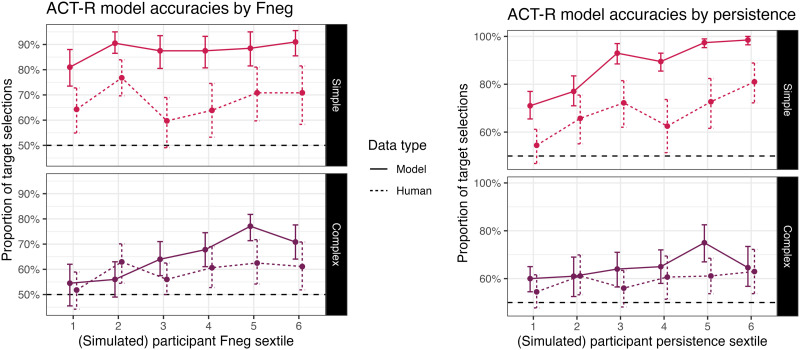
Comparing marginal individual difference patterns in our ACT-R model and human accuracy data for *F*_NEG_ (left), and persistence (right). Human data, represented here by dotted lines, is repeated from Figure 13.

The most important mismatches of this model lie largely in the magnitude of its expected effects: it overestimates overall accuracy, adaptation strength, and now we see that it also overestimates the predictive relationship between individual differences and accuracy (Figure 14). Even when fit to this particular dataset, the model reconstructs a strong *F*_NEG_ effect, when only a marginal relationship with our measure for that construct was attested, and a much stronger persistence relationship on top of this. Interestingly, these problems are not uniform across conditions: we can see in Figure 14 that average accuracy is well-modeled in complex trials, but far overestimated in simple trials, whereas the individual difference effects seem to be appropriately modeled in simple trials (notice that the model recreates a relatively minimal effect of *F*_NEG_ and a stronger effect of persistence) but overestimated in complex trials. In general, it seems that the particular relationship between simulated participants’ performance on simple and complex trials enforced in the current ACT-R model does not allow for a closer fit; this could be adjusted in some ways, see Discussion below.

Systematic comparison with other models can help us determine whether these cases of misalignment are enough to prefer alternative treatments of these patterns. We repeat the LOO model comparison procedure outlined above, fitting alternative simplified ACT-R models (A2, A3)^26^ and two heterogenous RSA models (B1, B2), and evaluating which model is best at generalizing to unseen data. As above, our full ACT-R model is a clear improvement over the other options tested (Table 7). The simplified alternative where *F*_NEG_ is fixed is a useful comparison point to establish whether the hypothetical explanatory value of *F*_NEG_ variance in the accuracy data is enough evidence to include the contributions of both individual differences in the model; unsurprisingly, this model receives second rank, but remains far behind the predictive power of the full model.

**Table 7.** Comparing model fit in terms of expected log predictive density (ELPD) estimates, between our full model and several alternatives.

**Model**	${\hat{\text{ELPD}}}_{LOO}$	**Std. Error**
Our Model	−2523.97	82.02
(A2) ACT-R with Fixed *F*_NEG_	−2678.35	74.93
(B1) Three-Type RSA	−3124.59	21.35
(B2) Flexible Three-Type RSA	−3128.39	20.86
(A3) Fully-Fixed ACT-R	−3227.52	33.14

### Discussion

The results analyzed above support several assumptions inherent in our adaptive approach here. First, we observe robust adaptation effects: Participants improve throughout the experiment, more quickly on the simple than on the complex trials, indicating a plausible role for reinforcement learning in the performance of this task. Second, our first considerations of response time data for this task reveal that participants do spend more time on critical trials than on unambiguous trials, suggesting that pragmatic reasoning in this game setting does involve some degree of additional effort.

The data also support some of the most important patterns predicted by our model, concerning the link between individual differences in the reference game task and parameters affecting resource-rational adaptation. In particular, participants with higher persistence scores also selected more pragmatic targets in the reference game. We also observe a trend of a positive effect of negative feedback strength (*F*_NEG_), and a trend of interaction of persistence and condition, where simple trials benefit more from persistence than complex ones. These trends are supported by the finding that our model generating accuracy distributions assuming variation in both *F*_NEG_ and persistence parameters still outperforms simpler alternatives, specifically including one which eliminates *F*_NEG_ variation. Although these results are not uniformly conclusive, they provide some evidence that the variability observed in reference games may emerge from general parameters governing task adaptation, unrelated to social reasoning. It may be the case that a domain-general measure of *F*_NEG_ is not as appropriate as a domain-general measure of persistence, although we do not take our results to rule it out entirely. It may equally be the case that there are other important aspects of variation in resource-rational adaptation which we do not measure here. In all, we take the positive result for persistence as an indication that at least some of the variation to be explained here can be linked to variation in domain-general aspects of task performance.

Still, in some places, this data interestingly mismatches the predictions of the particular model we have investigated above. Before moving on, we linger on these mismatches in predictions about responses, and response time, and frame what we can learn from them.

#### Mismatches in Response Patterns.

Given the focus of the present study on individual differences, it’s notable that our model ultimately overestimates the differences in response behavior that can be attributed to *F*_NEG_ and persistence. (Because we are comparing the effects of scaled predictors, we can interpret this as overestimating either underlying *F*_NEG_ and persistence effects, or the degree of variation in the population.) We consider here three possible explanations for this divergence.

One possibility is that our model fundamentally over-attributes the role of one or both of these particular factors in on-task adaptation. The model simulates a very particular form of strategy selection: low *F*_NEG_ traps a comprehender in a loop where they must repeat preferred but failing strategies multiple times before they might ever disengage and sample another more successful strategy. It is certainly possible that failures to identify successful pragmatic interpretation strategies do not actually come from problems disengaging, but some other difficulty in exploration which requires time to overcome. For instance, perhaps when a first-order or second-order interpretation strategy is considered for the first time, it takes time to correctly identify which information needs to be accessed and compared. In any such case, the effect of *F*_NEG_ would be somewhat weaker than is modeled here, while persistence would still play an important role. Such a model would be one way to explain why we observe a robust predicted effect of persistence, but not *F*_NEG_.

However, it is in fact both the *F*_NEG_ and the persistence effect that seem to be overestimated in our models. In light of this, another possibility is that this model is over-indexing on these two predictors because other important sources of variance are not accounted for. Even with the current task model, we would predict other variation dependent on individuals’ baseline willingness to consider more complex pragmatic interpretations—as partially modeled in Duff et al. (2025)—and a variety of cognitive factors which would control the speed and success rates of the strategies, like general processing speed, differences in working memory storage and retrieval, and differences in executive control and attention. Attributing some of the variation in behavior to these sources would reduce the degrees of variation our models currently assume in *F*_NEG_ and persistence.

A final explanation comes from more closely investigating where model predictions diverge from the patterns in our experiment. These divergences are not uniform; the model is sometimes able to fit participants’ parameter-dependent adaptation in one condition, but not another (cf. Figure 14). This suggests that the model may be specifically misguided in the relationship it assumes between between population-level success on simple trials and on complex trials. In our model as we have tested it here, if some participants have strong enough adaptation to achieve success on complex trials, and others do not, there must have been a wide distribution of participants, with the majority at some relatively high intermediate degree of success. That would expect lots of variation attributable to individual parameters in both conditions, and simple accuracy should overall be quite high, as we see in the predictions. But in reality, there seems to have been quite some distance between a cluster of participants who performed well in both conditions, and a larger cluster of participants who performed poorly in both conditions (Figure 11), even after we incorporated design elements to encourage task engagement. There are a number of ways that we could adjust the model to permit such discontinuity, including by stipulating multi-modal hyper-distributions of *F*_NEG_ or persistence, ^27^ or by building in other interacting sources of individual variation as described above. These solutions would, we expect, also alleviate the overestimation of individual difference effects, as well as overestimation of adaptation effects and general accuracy.

#### Mismatches in Response Time Patterns.

Response time data collected here highlighted one other notable misalignment between our model and behavior, regarding the comparison between simple and complex trials. Our model predicted, unintuitively, that correct responses would on average come slightly earlier in complex trials, due to a larger contribution of lucky guessing within the correct response sample in complex trials. In fact, human response times do suggest that guessing may contribute many faster response times in this condition—the lower peak in Figure 10 (right) would seem to be somewhat comparable to that in Figure 8—but their contributions aren’t enough to offset a larger increase in response time in that condition, compared to simple trials. Intuitively, it makes sense that correct responses should be slower in complex trials because the second-order interpretation participants must deploy there for non-chance accuracy is slower than the first-order interpretation which is sufficient in simple trials. Our model agrees that second-order reasoning should take longer, but this alone does not create substantial differences in average response times. This is because in our model, any subject who has begun to implement second-order reasoning in complex trials will also be selecting it for simple trials—the available task strategies are restricted to global preferences for literal, first-order, or second-order reasoning. We can imagine an additional set of strategies that may be plausibly adapted in this task, strategies involving the conditional execution of reasoning types. For instance: in our model as-is, optimal adaptation leads an agent to prefer strategy (C) in all cases when automatic application of (A) fails. Strategy (B), although it is more efficient than (C) in simple trials, should become universally dispreferred because of its low performance in complex trials. However, agents might also learn to deploy a more complicated strategy which was not modeled here, attempting to recognize trials where (B) is sufficient before falling back to the more-costly (C). Some evidence for this type of conditional deployment comes from the exploration of implicit negation by Misyak et al. (2016), who observe that interlocutors who use this unlikely reasoning option do so most often in cases where other, simpler options are insufficient. While implementing such conditioned execution as a strategy is non-trivial, we suggest that this approach might help explain the response time asymmetry in the human data: a participant following a conditional strategy would demonstrate a more robust pattern of slower responses in complex trials than in simple trials. Conditional strategies could also help explain the rare case where participants find success in complex trials but not simple trials (Figure 11). We leave deeper investigation of such strategies for future work.

## GENERAL DISCUSSION

We have tested here the hypothesis that comprehenders’ behavior in reference games is the outcome of resource-rational deployment of social reasoning. Operationalizing this proposal in ACT-R, we have shown in a simulation that flexible selection among strategies, guided by goals of efficient success, could explain why and when participants diverge from model predictions for rational second-order comprehension. In particular, we have highlighted a novel prediction of this approach: individual differences in performance on these tasks could come not only from variation in task-relevant competences, like social reasoning, but also from variation in the dynamics of task adaptation. The results of our experiment partially validate this prediction, and we conclude that individual differences in adaptation—like individuals’ persistence, their willingness to devote time and resources to a self-driven search for a reasonable interpretation—may indeed be responsible for the correlation observed in Mayn and Demberg (2026) between reference game behavior and performance on Raven’s Matrices.

As a result, although participants in reference games do not behave as orderly Gricean comprehenders, we are not compelled to reject the idea of Gricean pragmatic competence. Instead, we argue that we can see avoidance of second-order reasoning, effects of task exposure, and particular patterns of individual differences as a natural consequences of humans learning to deploy that competence effectively in an unfamiliar task.

In order to reach this conclusion in a convincing way, it was necessary to examine in detail the predicted behavior for a resource-rational participant, using insights from both our theories of competence and general models of task adaptation. Computational modeling of realistic, real-time processing behavior was instrumental in achieving this goal. For instance, the specificity and testability of a computational model’s predictions helps guard against a major obstacle in developing non-trivial models linking competence with performance. These models introduce so many new opportunities for flexibility at the level of performance that it could become hard, or even impossible, to disprove a competence theory. Indeed, in our case, one might wonder whether there is any evidence of non-Gricean behavior that one couldn’t explain away as a performance factor. By specifying a model that predicts only a particular type of non-isomorphic relationship between competence and observed behavior, and ties that relationship to patterns of behavior in unrelated tasks, we are confident that we are not at risk of this over-flexibility.

In developing a real-time model of performance in this task, we have also identified several predictions that push beyond participants’ response behavior towards questions of timecourse. We have already been able to offer an initial investigation of one set of these predictions, regarding response time distributions across conditions; some, but not all of the model predictions here are borne out. But our model also generates predictions about the distribution of visual attention across the display of our task, within each trial, and also differing across conditions and across exposure. These predictions remain to be tested, but they offer an opportunity to further evaluate and improve our approach, one which would not be possible without a clear view of how information is gathered and referenced across time in this task.

As we move towards better models of real-time pragmatic processing, we want to draw attention to important choices about the way competence is defined in these models. Our performance model in the end includes only a very simple approach to competence: the output of the literal, first-order, and second-order reasoning processes that we define sometimes differs from a complete Rational Speech Act model. For instance, our procedures are functions only of the feature distribution among the referents and messages in context. None of the procedures takes into account any differences in prior probability or salience across the referents, although these are known to help predict participant behavior, even for similarly simple stimuli, at least in one-trial tasks (Frank & Goodman, 2012; Qing & Franke, 2015; Sikos et al., 2021a). As we consider how gradience of this kind could be incorporated into our model, we note that it depends on another set of branching pathways for a mechanistic model of pragmatic performance: whether to account for stochasticity in competence or performance. One could define procedures of pragmatic interpretation in this task which are themselves non-deterministic: for instance, processing trial properties to estimate quality of all possible speaker referents, but then sampling among the referents weighted by quality, or selecting the best referent after application of some noise. On this kind of fundamentally-probabilistic approach, variation in a single participant’s behavior across similar trials can come from these competence-internal sources of stochasticity. In our approach, we took the second path, defining interpretation procedures which are themselves deterministic, but generating variation from differences in which procedure is applied, or mechanistic failures where a procedure is not applied appropriately. On the one hand, our model was not intended to rebuke the idea of a stochastic competence; we avoid it here for the practical reason that ACT-R does not lend itself to non-symbolic computation inside defined procedures, which seems necessary in this class of model. On the other hand, we observe that our model can predict appropriate within-participant variance as the result of noisy, variable performance, outperforming the probabilistic alternatives we tested. Future work aiming to use variable behavior as evidence for a fundamentally probabilistic pragmatic competence may benefit from comparisons against a baseline like this.

Regarding the generalizability of our current approach, we also note that we kept our focus here to a very simple task, which we assume is novel to our participants. In fact, it may seem at first glance that the role for resource-rational adaptation here is dependent on that novelty. It could be that in more conventional venues for pragmatic interpretation, like unconstrained conversation, experience leads participants to approach an interaction already defaulting to Gricean pragmatics, so that slower task adaptation would not stand in the way of drawing an inference. On the other hand, as Hawkins et al. (2021) point out, a resource-rational model also leaves room for another state of affairs, where the balance of interpretive value and resource demands means that in new interactions, we are still generally inclined to avoid costly applications of Gricean competence. Where a given person’s default behavior lies, on this account, should depend on their particular expectations about the utility of deeper reasoning in a given venue. It is therefore possible that these expectations, and differences in their adaptation, could underlie individual differences in the recognition of even conventional pragmatic meaning in text (Antoniou et al., 2016; Bott & Noveck, 2004; Fairchild & Papafragou, 2021; Heyman & Schaeken, 2015).

Differences in exploration may also be relevant for the derivation of less rigid pragmatic meaning, in a slightly different way. We have framed critical reference game trials as launching a search to reconstruct intention when literal interpretation does not yield an plausible explanation for observed behavior—for instance, when a literal interpretation does not meet typical expectations for informativity. This framing can be applied more generally to other *ad hoc* implicatures. When we consider communicative contexts which are more realistic, the search space for interpretive strategies expands from the set we have used here, differing only in recursive complexity, to a set varying in all sorts of parameters. Speakers may initially appear underinformative or overinformative even after the kinds of calculations considered here, possibly because they have different prior knowledge, possibly because they expect their interlocutor to have different prior knowledge, possibly because their conversational goals are simpler or categorically different, and many other potential hypotheses for a comprehender to consider (see Cummins, 2025 for a broader discussion along the same lines). This might be why Ryzhova et al. (2023) find a similar correlation between Raven’s Matrices performance and a particular *ad hoc* inference from overinformative utterances. In their study, participants read passages where a speaker described another character’s behavior, explicitly asserting that they performed an action which should have been trivially predictable in context (e.g., “They paid the cashier,” when describing a friend who bought groceries). Participants with higher performance on Raven’s Matrices were more likely to draw an “atypicality inference” in these cases, interpreting that this action would not usually be inferred because the character in question does not usually behave in the trivially predictable way (Kravtchenko & Demberg, 2022). An expanded version of our current model intuitively could predict this as a consequence of greater speed in exploring the wide space of other possible explanations for this overinformativity. We aim to extend the model towards this kind of data in our own future work.

## ACKNOWLEDGMENTS

The authors thank Natalia Bila for her assistance in creating the Anagram Persistence task, and Kata Naszadi, Michael Franke, Adrian Brasoveanu, Jakub Dotlačil, and Niels Taatgen, as well as audiences at MathPsych/ICCM 2024, XPrag.it 2024, and CogSci 2025 for helpful insights, discussion, and feedback. Robert Hawkins and three anonymous reviewers at Open Mind were instrumental in shaping the final form of this work.

## FUNDING INFORMATION

This project has received funding from the European Research Council (ERC) under the European Union’s Horizon 2020 research and innovation programme (ERC Starting Grant “Individualized Interaction in Discourse”, grant agreement No. 948878).

## AUTHOR CONTRIBUTIONS

J.D.: Conceptualization; Formal analysis; Investigation; Methodology; Software; Visualization; Writing – original draft; Writing – review & editing. A.M.: Conceptualization; Investigation; Methodology; Writing – original draft; Writing – review & editing. V.D.: Conceptualization; Funding acquisition; Supervision; Writing – review & editing.

## Data Availability Statement

The code for our ACT-R model, and the data and analysis scripts from our simulation and our experiment, are accessible in an OSF repository, available at https://osf.io/7uwx9/overview.

## Notes

^1^ We retain this term from previous work, although note that consideration of this object is critical for correct performance in “complex” trials.^2^ The amount of “greediness” at each layer can be adjusted in the model, through *α*, the inverse temperature parameter of a softmax function, often held at a constant moderate value. RSA predictions approach deterministic behavior as *α* approaches ∞.^3^ This downward revision in the number of first-order comprehenders matches the finding in Mayn and Demberg (2023) that the Franke and Degen (2016) stimuli in the simple condition were prone to unrelated biases that inflated target selections.^4^ For discussion on the ideosyncratic qualities of this temporal-difference variant, which is applied for utility estimation in ACT-R and is fundamental to our simulations below, see Brasoveanu and Dotlačil (2021, p. 474).^5^ Disengagement ability has elsewhere been suggested as an important domain-general cognitive construct (Shipstead et al., 2016; Storm et al., 2011). See Stocco et al. (2017) for a similar connection between *F*_NEG_ and attentional inhibition.^6^ This order is arbitrary, and adopted for simplicity. In fact, it’s likely that order of scanning is subject to random and by-participant variation, but without eye-tracking or response time data, this is as of yet uncertain.^7^ This is a simplified approach that works for all of the cases we consider. Because there are only two features for each of three possible referents, uniquely-good responses are always maximally-probable under one of the three strategies. To extend our model for games with additional features or referents, we would need to adopt more complicated methods for comparison.^8^ The model attempts memory retrieval of the first message in the bank, which may succeed if it has attended the bank recently. If it succeeds, it will continue evaluation using memory retrieval. If at any point, retrieval fails, it will revert to a visual scan to refresh memory.^9^ In fact, it is not exactly correct to say that Strategies (B) and (C) can solve unambiguous control trials. For instance, if there is only one referent which matches the observed message, but that referent has another matching message, strategy (B) as implemented here will not accept it as a maximally-probable referent. This loophole never occurs in practice, because strategy (A) is always attempted first, see Discussion below. The loophole itself is just a product of the hack to avoid comparison described in footnote 7: if true comparison were used, strategy (B) would recognize that this matching referent is still a better response than the alternatives.^10^ The units of *F*_NEG_ values are only interpretable in conjunction with learning rate and initial utility values, see below. Persistence values are integers corresponding to the standard internal temporal units of ACT-R, which map non-linearly to real-time units as described in Taatgen et al. (2007).^11^ The gulf in utilities may be taken to reflect a low expected value for pragmatic comprehension in a novel communication game, especially given the substantial extra time and effort these strategies require. We can use the particular utility learning equation in pyactr to relate these utilities to the conditions under which a production rule would approach them. To demonstrate for one parameterization, take 5 as the strength of positive feedback, and −5 as the strength of negative feedback; the length of the temporal offset between rule selection and reward is subtracted from these to yield the discounted reward. A utility of 5 reflects an (impossibly optimistic) rule with a 100% success rate and a 0 s delay. A utility of −5 could correspond to e.g., a strategy with a 100% success rate and a 10 s delay or a 50% success rate and a 5 s delay.^12^ Except the threshold, lowered to −1.0 to allow memory retrieval to be more frequently successful, and strength of association, set to 3.2 to ensure activation boosts were only ever positive. We also adopted a standard small value for the activation noise parameter, 0.15.^13^ This was a simplifying assumption; in principle these parameters may be correlated in the population. As it happens, the measures of these constructs from our human-subjects experiment below suggest that independence is plausible.^14^ These distributions were truncated at the boundaries of the parameter values under consideration in order to map to simulated ACT-R participants; therefore they become effectively half-normal distributions as their centers approach the edges.^15^ These 228 are the subset of participants in their dataset who passed exclusion criteria for the reference game and had not taken part in a Raven’s-like test in the past six months.^16^ The latter is a smaller increase on the logistic scale, as diagnosed by a notable interaction between trial and condition.^17^ Besides correctly and quickly applying strategy (C), or quickly guessing after an attempt at strategy (A), other ways to obtain a correct answer in a complex trial include lucky guesses after single attempts at strategy (B), lucky guesses after chains of multiple attempts of strategies (A) and (B), and chains of one or more attempts of strategies (A) and (B) followed by a successful application of strategy (C).^18^ In simple trials, 20.53% of correct responses came from chains of one to four attempts at (A) followed by a guess, all of which were faster than the execution time for (B) or (C). In complex trials, where the overall number of correct responses fell, the same cases made up 28.10% of correct responses, although they had a lower absolute frequency.^19^ Of course, these cases also featured fewer cycles of strategy application, but what this shows us is that response time in our simulation was more often determined by the complexity of the participant’s preferred strategy than by willingness to attempt multiple strategies. Indeed, on inspecting simulation traces, the majority of correct answers to simple or complex trials came after just one application of strategy (B) or (C).^20^ A reviewer points out another set of interesting predictions regarding performance under time constraints. As a consequence of differences in execution time, and the influence of multiple attempts in supporting adaptation, our model expects time constraints to reduce both accuracy and adaptation. We hope future work can investigate this prediction.^21^ Once participants had completed all trials in the main body of the task, they saw one simple and one complex trial again, in that order. In each case, after they had made their selection, participants were prompted to explain why they made that decision in a textbox, as in Mayn and Demberg (2023). We plan to annotate participants’ reported strategies for future analyses of this data.^22^ See Appendix D for a post-hoc analysis of only those participants who passed the criterion. This smaller sample exhibits similar basic patterns, but fails to recover any predicted individual difference effects, which we attribute to the sharply reduced sample size.^23^ Marginal contrasts from model estimates reveal a 95% HDPI of [0.02, 0.05] for the trial effect in simple trials, and [0.00, 0.02] in complex trials.^24^ As a reviewer notes, this is perhaps not as robust a correlation as we might expect. Indeed, Dale et al. (2018) report correlations of above *r* = .50 between skipping-time-based metrics for several impossible Raven’s problems and several impossible trials of another task. We think it is possible that our lower correlation here is due to reduced reliability of our Raven’s measure, as it was extracted from only one impossible trial.^25^ A reviewer raises another more specific way of framing this effect: as we know interacting dyads generally become smoother communicators over time (Brennan & Clark, 1996, among many others), it is possible comprehenders are attempting to settle into fixed patterns of message encoding and decoding. While this cannot be as simple as establishing referential pacts for specific objects, as objects occur many times with both their possible messages, we agree that we can think of this as a similar type of conversation-specific alignment. Whether this is best modeled as increasingly automatic comprehension procedures vs. something more conscious remains an interesting open question.^26^ We do not report LOO values for (A1), the fixed-persistence model, because estimation failed reliability diagnostics. A high Pareto-$\hat{k}$ diagnostic indicated that fits for this model were very sensitive to the inclusion of specific observations, which causes problems for reliable ELPD estimation. This is usually thought to result from an especially-poorly-specified model which cannot generalize appropriately (Vehtari et al., 2017). Comparing the model trace on this dataset to the (relatively unproblematic) trace on the Mayn and Demberg (2026) data, we note that just in this dataset, chains sometimes struggled to distinguish between fits with a low fixed persistence and widely-varying *F*_NEG_, vs. a higher fixed persistence with a lower, less varying *F*_NEG_. This two-way uncertainty may be responsible for particular predictive instability. Note that if we set aside the $\hat{k}$ diagnostic, our best estimate of ${\hat{ELPD}}_{LOO}$ would place this model above (B1) but below (A2) and our full model.^27^ There is some evidence for bimodality already in our PSS measure of *F*_NEG_, although not for our Anagram Persistence measure.

## Figures and Tables

**Figure C.1.**
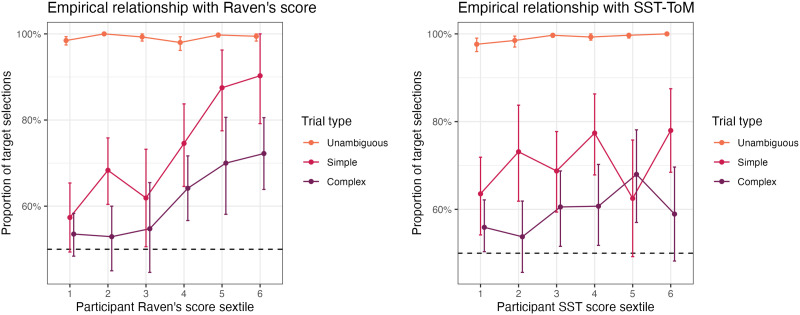
Marginal relationships observed for reference game accuracy with Raven’s score (left), and with Short Story Task-derived Theory of Mind score (right).

**Figure C.2.**
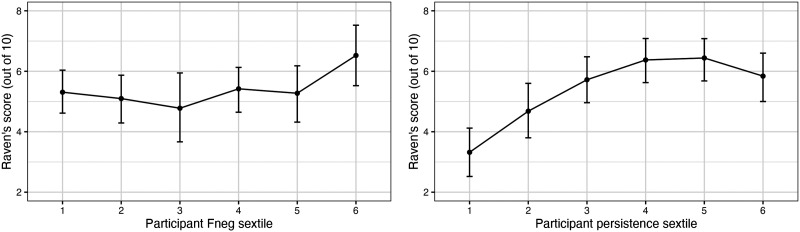
Marginal relationships for Raven’s score with Probabilistic Stimulus Selection-derived *F*_NEG_ (left) and with anagram persistence (right).

## APPENDIX A: MORE DETAILS ON REGRESSION ANALYSES FOR SIMULATIONS AND EXPERIMENTS

### Our simulation of Mayn and Demberg (2025)

After fitting hyperparameters for our model against the response data from Mayn and Demberg (2025), and extracting a simulated sample, we fit a Bayesian logistic regression to the responses in that sample using the R package brms (Bürkner, 2017). This regression examined the effects of condition, trial number, *F*_NEG_, persistence, and their various first-order interactions; we describe effects as notable if 95% highest-density posterior intervals exclude 0. The regression was only fit to data from simple and complex trials; the condition predictor was thus binary, sum-coded with complex trials as +1. The trial predictor was centered, and *F*_NEG_ and persistence were scaled and centered. Wide regularizing priors were included for all predictors, informed by previous empirical results. While we present here only one sample, repeated sampling yielded very similar values for posterior estimates. Resulting parameter posteriors are presented in Table A.1.

**Table A.1.** Logistic mixed-effect regressions for accuracy from our simulated sample modeled on Mayn and Demberg (2025), and Mayn and Demberg (2025) itself (where variables were comparable). Contrast coding directions are listed as (negative vs. positive). Effects for which the 95% HDPI does not include 0 are **bolded**.

**Effect**	**Simulated Data**		**Mayn and Demberg (2025) Data**	
	**Estimate**	**95% HDPI**	**Estimate**	**95% HDPI**
Intercept	**1.32**	[1.23, 1.41]	**1.32**	[1.08, 1.56]
Condition (simple vs. complex)	**−0.81**	[−0.89, −0.72]	**−0.45**	[−0.61, −0.30]
TrialNumber	**0.03**	[0.02, 0.03]	**0.19**	[0.04, 0.35]
Condition: TrialNum	**−0.01**	[−0.01, −0.01]	0.14	[−0.00, 0.28]
*F*_NEG_	**0.65**	[0.56,0.74]		
Persistence	**0.77**	[0.68, 0.86]		
Condition: *F*_NEG_	**−0.12**	[−0.20, −0.04]		
Condition: Persistence	**−0.45**	[−0.53, −0.37]		
TrialNum: *F*_NEG_	**0.01**	[0.01, 0.02]		
TrialNum: Persistence	**0.01**	[0.01, 0.01]		
*F*_NEG_: Persistence	**0.29**	[0.21, 0.37]		

We also fit a Bayesian log-normal regression to simulated response time in milliseconds, subsetting only to trials with correct responses, examining the effects of condition, trial number, *F*_NEG_, persistence, and their various first-order interactions. In this case, the regression was fit to unambiguous filler trials as well as simple and complex trials; the three-way condition predictor was Helmert-coded to compare unambiguous vs. critical, and simple vs. complex. Other contrasts were coded as in the accuracy model. Resulting parameter posteriors are presented in Table A.2.

**Table A.2.** Log-normal mixed-effect regressions for response time from our simulation. Contrast coding directions are listed as (negative vs. positive). Effects for which the 95% HDPI does not include 0 are **bolded**.

**Effect**	**Estimate**	**95% HDPI**
Intercept	**7.817**	[7.813, 7.821
Condition1 (unambig. vs. critical)	**0.289**	[0.287, 0.292]
Condition2 (simple vs. complex)	**−0.025**	[−0.030, −0.019]
TrialNumber	**0.003**	[0.003, 0.004]
Condition1: TrialNum	**0.002**	[0.002, 0.002]
Condition2: TrialNum	**0.000**	[0.000, 0.001]
*F*_NEG_	**0.067**	[0.063, 0.071]
Persistence	**0.125**	[0.121, 0.129]
Condition1: *F*_NEG_	**0.033**	[0.030, 0.035]
Condition2: *F*_NEG_	**0.006**	[0.001, 0.012]
Condition1: Persistence	**0.063**	[0.061, 0.065]
Condition2: Persistence	**0.009**	[0.004, 0.014]
TrialNum: *F*_NEG_	0.000	[−0.000, 0.000]
TrialNum: Persistence	**−0.001**	[−0.001, −0.001]
*F*_NEG_: Persistence	**−0.013**	[−0.016, −0.010]

### Population-Level Patterns in the Present Experiment

To determine whether participants as a whole exhibited patterns of adaptation and conditional differences in accuracy observed in previous studies, we fit a Bayesian logistic mixed-effect regression to the accuracy data. Correctness of the selection on a given trial, a binary variable, was regressed onto condition (Helmert-coded, unambiguous fillers vs. critical, and simple vs. complex), trial number (mean-centered), message type (sum-coded, −1 = shape, 1 = color), target position (left, center or right, dummy-coded with center as reference), and the interaction between trial number and condition. Message type and target position were included as covariates following previous studies. The random effect structure was maximal, including a per-participant random intercept and per-participant random slopes for condition, trial number, interaction between condition and trial, message type and target position. We did not include random effects for item, as item identity had no meaningful differences not accounted for by covariates.

Wide regularizing priors were included for all predictors, biasing towards realistic effect sizes congruent with previous observations in similar studies (Franke & Degen, 2016; Mayn & Demberg, 2023, 2025). For both models, we ran 6 chains for 5000 iterations, the first 500 of which were discarded as warmup.

As in our analysis of our simulated data, to assess the evidence for the effects we examined the 95% highest-density posterior intervals of the parameter estimates. If these intervals excluded zero, we concluded that there was a notable relationship between the predictor and the dependent variable. Resulting parameter posteriors are presented in Table A.3.

**Table A.3.** Mixed-effect regressions for accuracy (logistic) and response times (log-normal). Contrast coding directions are listed as (negative vs. positive). Effects for which the 95% HDPI does not include 0 are **bolded**.

**Effect**	**Model of accuracy**		**Model of RT**	
	**Estimate**	**95% HDPI**	**Estimate**	**95% HDPI**
Intercept	**2.73**	[2.39, 3.14]	**7.72**	[7.64, 7.81]
Condition1 (unambig vs. rest)	**−1.73**	[−2.07, −1.46]	**0.06**	[0.04, 0.08]
Condition2 (simple vs. complex)	**−0.40**	[−0.56, −0.26]	0.01	[−0.01, 0.03]
TrialNumber	0.01	[−0.01, 0.02]	**−0.02**	[−0.02, −0.02]
MessageType (shape vs. color)	0.01	[−0.09, 0.11]	0.01	[−0.00, 0.02]
TargetPosition1 (center vs. left)	−0.18	[−0.48, 0.14]	0.00	[−0.01, 0.02]
TargetPosition2 (center vs. right)	−0.19	[−0.46, 0.10]	−0.01	[−0.02, 0.01]
Condition1: TrialNumber	0.02	[−0.00, 0.03]	−0.00	[−0.00, 0.00]
Condition2: TrialNumber	−0.01	[−0.02, 0.00]	0.00	[−0.00, 0.00]

We also fit a Bayesian log-normal regression with the same predictors as for the accuracy model to response time on correct trials. No exclusion criterion based on RT was preregistered, as we didn’t anticipate extreme outliers. Nevertheless, some trials had response times of above 20 s. We conducted a secondary analysis of RT excluding these cases, and it was consistent with the reported model in all important respects, with only minor numerical differences. As such, we have reported the analysis on the full dataset.

### Individual Differences in the Present Experiment

We then fit Bayesian regression models which built on the models described above and additionally included critical measures for *F*_NEG_ and persistence. For these models, we exclude the unambiguous trials and keep only the critical trials, because unambiguous trials were used as a baseline for understanding the task and we do not predict a relationship with accuracy with individual differences there.

The regression models included the same predictors as the models reported in the previous section, as well as *F*_NEG_ and anagram persistence, centered and scaled, and interactions of *F*_NEG_ and persistence with condition, with trial order, and with each other. Because unambiguous trials were dropped for this analysis, condition was recoded to use sum-coding with complex trials as +1.

Resulting parameter posteriors are reported in Table A.4. A few notes on the effects that were not particular to individual differences: Like in the analysis without individual differences, this analysis supports the conclusion that participants are less accurate on the complex trials than on the simple ones. We also find that accuracy in these trials improved over the course of the experiment. Note that this is consistent with the model without individual differences, where marginal effects showed that people’s performance increases more on the critical trials. Unlike the previous analysis, this analysis also finds evidence for people’s performance improving differentially on the two critical conditions: it improves less strongly in the complex condition than in the simple condition.

**Table A.4.** Mixed-effect regressions for accuracy (logistic) and response times (log-normal), with *F*_NEG_ and persistence as predictors. Contrast coding directions are listed as (negative vs. positive). Effects for which the 95% HDPI does not include 0 are **bolded**.

**Effect**	**Model of accuracy with IDs**		**Model of RT with IDs**	
	**Estimate**	**95% HDPI**	**Estimate**	**95% HDPI**
Intercept	**1.05**	[0.81, 1.31]	**8.12**	[8.03, 8.22]
Condition (simple vs. complex)	**−0.40**	[−0.55, −0.25]	**0.02**	[0.01, 0.04]
TrialNumber	**0.02**	[0.01, 0.03]	**−0.02**	[−0.02, −0.01]
MessageType (shape vs. color)	−0.00	[−0.10, 0.10]	0.01	[−0.00, 0.03]
TargetPosition1 (center vs. left)	−0.22	[−0.55, 0.12]	0.00	[−0.02, 0.02]
TargetPosition2 (center vs. right)	−0.21	[−0.48, 0.07]	−0.00	[−0.02, 0.01]
Condition: TrialNum	−0.01	[−0.02, 0.00]	0.00	[−0.00, 0.00]
*F*_NEG_	0.17	[−0.05, 0.40]	0.00	[−0.02, 0.02]
Persistence	**0.32**	[0.08, 0.58]	0.00	[−0.02, 0.02]
Condition: *F*_NEG_	−0.04	[−0.19, 0.11]	0.00	[−0.01, 0.02]
Condition: Persistence	−0.15	[−0.32, 0.02]	0.00	[−0.02, 0.02]
TrialNum: *F*_NEG_	−0.00	[−0.02, 0.01]	−0.00	[−0.00, 0.00]
TrialNum: Persistence	0.00	[−0.01, 0.01]	−0.00	[−0.01, 0.00]
*F*_NEG_: Persistence	−0.01	[−0.31, 0.30]	0.00	[−0.02, 0.02]

The preregistered regression over response time featured several divergent transitions, diagnosing degeneracy in the fit. As preregistered, we simplified random effects until the regression was able to successfully converge; as a result, this regression featured only random slopes for condition, trial number, message type, and target position.

### Simulating Our Experimental Results

Complete regression parameters fit to a representative draw in terms of accuracy and RTs are reported in Table A.5.

**Table A.5.** Mixed-effect regressions for accuracy (logistic) and response times (log-normal) on a representative sample from the model simulation. Contrast coding directions are listed as (negative vs. positive). Effects for which the 95% HDPI does not include 0 are **bolded**.

**Effect**	**Model of simulated accuracy**		**Model of simulated RT**	
	**Estimate**	**95% HDPI**	**Estimate**	**95% HDPI**
Intercept	**1.66**	[1.52, 1.82]	**8.20**	[8.19, 8.22]
Condition (simple vs. complex)	**−1.01**	[−1.16, −0.86]	**−0.01**	[−0.02, −0.00]
TrialNumber	**0.06**	[0.05, 0.08]	**0.01**	[0.01, 0.01]
Condition: TrialNum	**−0.03**	[−0.04, 0.02]	0.00	[−0.00, 0.00]
*F*_NEG_	**0.52**	[0.40, 0.63]	**0.02**	[0.01, 0.03]
Persistence	**0.79**	[0.65, 0.94]	**0.12**	[0.11, 0.13]
Condition: *F*_NEG_	−0.13	[−0.25, 0.02]	−0.01	[−0.02, 0.00]
Condition: Persistence	**−0.52**	[−0.66, −0.39]	0.01	[−0.00, 0.02]
TrialNum: *F*_NEG_	0.02	[0.01, 0.03]	−0.00	[−0.00, 0.00]
TrialNum: Persistence	0.02	[0.01, 0.03]	**−0.01**	[−0.01, −0.00]
*F*_NEG_: Persistence	0.16	[0.07, 0.26]	0.00	[−0.01, 0.01]

## APPENDIX B: MORE DETAILS ON BAYESIAN MODEL-FITTING AND COMPARISONS

In this section, we provide more details on the general approach adopted to fit the hyper-parameters for our ACT-R simulations and alternative models.

In all cases, models were fit to maximize the likelihood of the log-odds of target accuracy in simple and complex trials for each quarter of the experiment, for each participant. While we do not generally see this approach as preferable to modeling likelihood at the single-trial level, we fit participant-level values in order to avoid the problem of handling auto-correlation within each subject, which would require hierarchical estimation of individual participant random effects. This degree of specificity would require gradient-free forms of Bayesian estimation, and models of this form did not converge.

Given the task of participant-level likelihood maximization, we were able to fit the important hyper-parameters while marginalizing over specific participant values. For instance, in our full model, this was achieved by using the density of the normal distributions around *F*_NEG_ and persistence to weight components of a mixture corresponding to the log-odds expectations for participants with those parameter values, where the center and spread of each component distribution was derived through repeated ACT-R simulation. Other ACT-R models (A1–A3) were fit with the same principles, but with negligible spread hyper-parameters for parameters that were fixed.

Models (B1) and (B2), RSA models generalizing Franke and Degen (2016), were also implemented as mixture models, in this case over the three unitary reasoning types, where the three weights were estimated directly. The center for each component distribution was uncertain, depending on expected distribution of the greediness parameter *α*, which was itself drawn from a Gamma hyperdistribution as in Franke and Degen (2016), whose shape and rate parameters *a_α_, b_α_* were estimated. For any *α, L*_0_, *L*_1_, and *L*_2_ expectations were deterministic results of the trial type, and the RSA calculations specified by Franke and Degen (2016). Note that in their specification for these comprehender types, in *L*_2_, *α* scales the greediness of the internal *S*_1_, rather than the comprehender itself, while it scales *L*_0_ towards accurate literal interpretation, and *L*_1_ towards complete *L*_1_ reasoning (rather than scaling the internal *S*_0_ towards literal message selection). A final hyperparameter, *σ* was applied to calculate some noise around the predictions of each comprehender type. Only in (B2), weights for the mixture were allowed to vary across quarters of the experiment. A more-complex alternative (B3) was considered where greediness hyper-parameters also varied by quarter, but it produced no notable differences and was left out for brevity.

All models were fit using NUTS in pymc with loosely constraining priors and 10000 draws. target_accept was raised above its default value of 0.8 in some models in order to address divergences. Tuning iterations were increased from a minimum of 1000 to a maximum of 5000 to address convergence difficulties.

In model-fitting, some models stubbornly resisted convergence (specifically, A2 and A3, for both the mayn2023 simulations and the simulations of our own dataset). Rather than discard them as ill-fitted, we report their performance as it stands, observing that in these cases, convergence failures occurred when chains identified separate but comparable optima, apparently stable states where these particular partially-fixed models made comparable predictions.

## APPENDIX C: ANALYSES OF ADDITIONAL INDIVIDUAL DIFFERENCE MEASURES

As reported briefly in our experimental results in the main body, in addition to investigating individual differences as a factor of *F*_NEG_ and persistence scores, we conducted additional analyses investigating the role of two additional tasks, Raven’s Matrices (Raven et al., 1998), and the Short Story Task (Dodell-Feder et al., 2013).

### Replicating the Relationships in Mayn and Demberg (2025)

In their sample of reference game performance, Mayn and Demberg (2025) report positive relationships (a) between accuracy and a construct for Theory-of-Mind, composed from scores on the Short Story Task (Dodell-Feder et al., 2013) and Reading the Mind in the Eyes (Baron-Cohen et al., 2001) and (b) between accuracy and a construct for reasoning ability, composed from scores on a shortened form of Raven’s Advanced Progressive Matrices (Raven et al., 1998) and the Cognitive Reflection Task (Frederick, 2005). The latter relationship, specifically with Raven’s, is predicted by our model of task adaptation, but the former relationship is also a standard prediction of any model that casts pragmatic inference via social reasoning. As a result, we included one representative from each of these constructs in our battery of tasks in the present experiment. While limiting ourselves to one task does reduce our ability to accurately assess a general task-independent construct, these relationships were not the primary interest of this study.

We first attempt to assess these relationships in isolation, as a simplified replication of the original analysis of Mayn and Demberg (2025). We fit a Bayesian logistic mixed-effect regression, predicting correctness of the selection on a given trial as a function of condition (sum-coded, −1 = simple, 1 = complex), trial number (mean-centered), message type (sum-coded, −1 = shape, 1 = color), target position (left, center or right, dummy-coded with center as reference), the interaction between trial number and condition, plus Raven’s score (mean-centered and scaled), SST score (mean-centered and scaled), and their interactions with condition. While we attempted to use a maximal random effect structure, due to the presence of divergent transitions, we simplified until we were able to find a well-behaved convergence; this regression featured random slopes for condition, trial number, message type and target position by subject. Resulting parameter posteriors are presented in Table C.1. See Figure C.1 for visualizations of the two key relationships.

**Table C.1.** Logistic mixed-effect regression for accuracy as a function of Raven’s and SST scores with standard covariates. Contrast coding directions are listed as (negative vs. positive). Effects for which the 95% HDPI does not include 0 are **bolded**.

**Effect**	**Estimate**	**95% HDPI**
Intercept	**1.03**	[0.80, 1.28]
Condition (simple vs. complex)	**−0.42**	[−0.58, −0.28]
TrialNumber	**0.02**	[0.01, 0.03]
MessageType (shape vs. color)	−0.00	[−0.11, 0.11]
TargetPosition1 (center vs. left)	−0.22	[−0.53, 0.11]
TargetPosition2 (center vs. right)	−0.23	[−0.52, 0.06]
Condition: TrialNum	−0.01	[−0.02, 0.00]
Raven’s	**0.47**	[0.27, 0.68]
SST	0.00	[−0.20, 0.21]
Condition: Raven’s	**−0.19**	[−0.34, –0.04]
Condition: SST	0.00	[−0.14, 0.15]

Together with expected effects of condition and trial number, we observe that we replicate a robust positive relationship with Raven’s scores, $\hat{\beta }$ = 0.47, modulated by condition, $\hat{\beta }$ = −0.19. Participants with higher Raven’s scores achieve higher performance on the reference game, and this produces the largest gains in accuracy in simple trials. This closely replicates the role of reasoning in Mayn and Demberg (2025).

Nevertheless, we do not observe a notable relationship with SST scores, $\hat{\beta }$ = 0.00. As noted in the main text, this is a striking departure from Mayn and Demberg (2025), but it could be explained as a factor of the changes we have made to the task here, in particular introducing feedback that may have raised the salience of the potential intentions of an imagined interlocutor, thereby levelling differences between those who generally would devote resources to imagining those intentions, and those who would not. We do not feel that this challenges the contributions of social reasoning to the generation of pragmatic inferences.

### Predicting Raven’s Matrices Performance

To explain the Mayn and Demberg (2025) association between Raven’s and reference game, we considered that individual differences in both tasks may be driven by variance in reinforcement learning parameters like *F*_NEG_ and persistence. For Raven’s, this argument was independently made by Stocco et al. (2021), who validate a dependence on *F*_NEG_, but the persistence effect which they predict has not been previously validated. Having collected Raven’s scores together with measures of *F*_NEG_ and persistence, we are in a position to examine whether both predictors indeed help predict Raven’s score.

To do so, we fit a Bayesian binomial regression, estimating the number of correct selections in the task out of a maximum of 10, as predicted by PSS-derived *F*_NEG_ and Anagram persistence (both centered and scaled), together with their interaction. Resulting parameter posteriors are presented in Table C.2. We observe notable positive effects of both *F*_NEG_, $\hat{\beta }$ = 0.14, and persistence, $\hat{\beta }$ = 0.34, as well as a superadditive interaction between them, $\hat{\beta }$ = 0.16. See Figure C.2 for visualizations of the two relationships.

**Table C.2.** Binomial regression for Raven’s Score as a function of *F*_NEG_ and persistence scores and their interaction. Effects for which the 95% HDPI does not include 0 are **bolded**.

**Effect**	**Estimate**	**95% HDPI**
Intercept	**0.17**	[0.07, 0.27]
*F*_NEG_	**0.14**	[0.03, 0.25]
Persistence	**0.34**	[0.22, 0.45]
*F*_NEG_: Persistence	**0.16**	[0.02, 0.30]

We note that this matches closely with the predictions of the Stocco et al. (2021) model for Raven’s, where both *F*_NEG_ and persistence contribute to success, each providing a mechanism for an agent to more likely disengage from rules that have been hypothesized but then rejected. Although it is not reported in Stocco et al. (2021), that model indeed predicts the superadditive interaction as well, as agents with high persistence will have more opportunities to sample hypotheses before guessing, and thus more occasions to benefit from a higher *F*_NEG_. We thus validate one of the key assumptions linking Raven’s and the reference game: both tasks benefit from parameters that allow for more rapid utility learning.

### Probing for Remaining Raven’s Effects

Given that we replicated the relationship between Raven’s and reference game performance from Mayn and Demberg (2025), and that we have shown that *F*_NEG_ and persistence are associated with Raven’s variance, we might wonder how much of the observed Raven’s–reference game relationship can be explained by a shared dependence on *F*_NEG_ and persistence. As one way to estimate this, we wanted to measure effects of Raven’s on residual variance in reference game accuracy which *F*_NEG_ and persistence were not able to explain. We first extracted randomized quantile residuals from the model reported in Table A.4, following the procedure described in Kay (2023), and then subjected these resiudals to an additional linear regression with centered scaled Raven’s score, SST score, and their interactions with condition as predictors. Posterior parameters are reported in Table C.3, on the left.

**Table C.3.** Linear mixed-effect regression over randomized quantile residuals from (left) the logistic regression reported in Table A.4, and (right) a version of the logistic regression reported in Table A.3 fit to only critical trials. Contrast coding directions are listed as (negative vs. positive). Effects for which the 95% HDPI does not include 0 are **bolded**.

**Effect**	**Residuals from ID model**		**Residuals from no-ID model**	
	**Estimate**	**95% HDPI**	**Estimate**	**95% HDPI**
Intercept	**0.01**	[0.00, 0.01]	**0.01**	[0.00, 0.01]
Raven’s	**0.03**	[0.03, 0.03]	**0.04**	[0.03, 0.04]
SST	**−0.01**	[−0.01, −0.00]	−0.00	[−0.01, 0.00]
Condition: Raven’s	**−0.01**	[−0.01, −0.00]	**−0.01**	[−0.02, −0.01]
Condition: SST	0.00	[−0.00, 0.01]	**0.00**	[0.00, 0.01]

We indeed recover a relationship between Raven’s scores and the residuals from our model, both a main effect, $\hat{\beta }$ = 0.03, and an interaction with condition, $\hat{\beta }$ = –0.01, such that higher Raven’s scores were associated with cases where our model underpredicted actual likelihoods of target selections, with larger underpredictions in simple trials than complex trials. We can conclude that the relationships with *F*_NEG_ and persistence estimated in our original regression did not fully subsume the observed relationship with Raven’s scores. Further comparison with the residuals from a model with no individual difference measures (Table C.3, right) allows us to check how much was subsumed: we can see that in the absence of *F*_NEG_ or persistence effects, Raven’s scores have a slightly stronger relationship with residuals, $\hat{\beta }$ = 0.04. Thus, we conclude that these two variables on their own only account for a small proportion of the Raven’s-related variance in reference game performance, leaving much of the association in need of further explanation. Of course, shared patterns in Raven’s and reference game performance may be contingent on a variety of other unmeasured aspects of task adaptation, and individual characteristics related to other domain-general mechanisms (efficient memory encoding and access, visual acuity, etc.). We leave it to future work to identify what those other characteristics may be.

Incidentally, the first residual regression also recovers a small negative relationship between SST scores and residuals from the *F*_NEG_-persistence model, $\hat{\beta }$ = –0.01. That is, it would appear our main regression tended to slightly overpredict the likelihood of target selections from participants with larger SST scores. This surprising effect is only observed in our model of residuals, and does not correspond to any available hypotheses about the role of Theory of Mind in pragmatic reasoning, so we consider for now that it may be an artifact of collinearity between individual difference predictors, and warrants further replication before any conclusions can be drawn. The same approach seems reasonable for the borderline interaction of condition and SST visible only in the second residual regression.

## APPENDIX D: ANALYSIS OF MAIN PATTERNS GIVEN STRICTER EXCLUSION CRITERIA

In our main analysis, we diverged from our preregistered exclusion criteria, including participants who did not successfully generalize the training patterns in the Probabilistic Stimulus Selection task after the maximum number of training block repetitions. Our logic for planning to exclude these participants was that, without evidence that they had learned to prefer stimulus A (which had a high frequency of reward) vs. stimulus B (which had a low frequency of reward), avoidance of stimulus B in other pairs in the test block may not provide a valid measure of negative feedback strength. Nevertheless, more than 40% of our participants failed to pass this criterion; given that it would have been prohibitive to ensure a large sample of participants who all passed, and that previous work had not used this exclusion criterion (Frank et al., 2004, 2005, 2007; Stocco et al., 2021), we in the end opted to include these participants in our main analysis.

To understand the consequences of this departure from the preregistration, we report here two supplementary analyses which examine the patterns in the subset of 92 participants that passed our original criteria. In Table D.1, we report posteriors over parameters from regressions for accuracy and response time, comparable to those reported in Table A.4. Due to divergent transitions, the random effect structure for the accuracy model had to be simplified, in the end only including a random intercept and random slopes for condition, trial number, message type, and target position. By analogy to the main analysis, the random effect structure in the RT model was simplified in the same way.

**Table D.1.** Mixed-effect regressions for accuracy (logistic) and response times (log-normal), with *F*_NEG_ and persistence as predictors, on the subset of the data from participants who passed the original PSS exclusion criterion. Contrast coding directions are listed as (negative vs. positive). Effects for which the 95% HDPI does not include 0 are **bolded**.

**Effect**	**Model of accuracy with IDs**		**Model of RT with IDs**	
	**Estimate**	**95% HDPI**	**Estimate**	**95% HDPI**
Intercept	**1.24**	[0.90, 1.61]	**8.20**	[8.10, 8.31]
Condition (simple vs. complex)	−**0.51**	[−0.72, −0.32]	0.01	[−0.00, 0.03]
TrialNumber	**0.03**	[0.01, 0.04]	−**0.01**	[−0.02, −0.01]
MessageType (shape vs. color)	−0.00	[−0.14, 0.14]	0.01	[−0.01, 0.03]
TargetPosition1 (center vs. left)	−0.33	[−0.75, 0.12]	0.00	[−0.02, 0.02]
TargetPosition2 (center vs. right)	−0.33	[−0.71, 0.08]	−0.01	[−0.02, 0.01]
Condition : TrialNum	−0.01	[−0.02, 0.01]	−0.00	[−0.00, 0.00]
*F*_NEG_	0.05	[−0.24, 0.34]	−0.00	[−0.02, 0.02]
Persistence	0.12	[−0.18, 0.42]	0.00	[−0.02, 0.02]
Condition: *F*_NEG_	−0.06	[−0.25, 0.12]	0.01	[−0.01, 0.02]
Condition : Persistence	−0.02	[−0.21, 0.16]	0.00	[−0.02, 0.02]
TrialNum: *F*_NEG_	−**0.02**	[−0.03, –0.00]	−0.00	[−0.01, 0.00]
TrialNum: Persistence	−0.00	[−0.01, 0.01]	0.00	[−0.00, 0.00]
*F*_NEG_: Persistence	−0.01	[−0.27, 0.26]	0.00	[−0.02, 0.02]

In our accuracy model, we observe expected effects of condition and trial, similar to those in the main analysis. Other effects do not appear clearly in this sample, including the interaction of condition and trial, and the critical effect of persistence—likewise, the trending effect of *F*_NEG_ is here much farther from our criterion for notability. One effect appears which we had not observed in the main analysis: a small interaction of *F*_NEG_ and trial, $\hat{\beta }$ = –0.02. Marginal comparisons across model posteriors reveal an estimated effect of trial is robust for participants with *F*_NEG_ one standard deviation below the average, but smaller for participants one standard deviation above the average, to the point that the HDPI for that marginal effect overlaps 0. Note that our model expects the reverse, that higher *F*_NEG_ should be associated with higher rates of adaptation.

The absence of any evidence for critical individual difference effects, and the presence of this unexpected interaction, would both seem to be strikes against the predictions of our model. Nevertheless, we suspect that the power reduction from looking at this smaller sample may place us at risk of both Type I and Type II errors. With no particular reason to expect that effects in the complete sample should have been solely due to the PSS-exclusion participants, and no particular reason to expect *F*_NEG_ to produce weaker adaptation, we find it best to engage with the patterns visible in the full analysis.

Along the same lines, the subset analysis of response times only repeats the finding of participants speeding up as they complete more trials of the task. Response times in complex trials are higher, but unlike in the main analysis, this effect falls short of our HDPI criterion for notability. We again do not take this as evidence against the conclusion presented in the main text, instead we simply consider that these effects are likely not reliably detectable with power below our planned sample size.

## References

[bib1] Anderson, J. R., Bothell, D., Byrne, M. D., Douglass, S., Lebiere, C., & Qin, Y. (2004). An integrated theory of the mind. Psychological Review, 111(4), 1036–1060. 10.1037/0033-295X.111.4.1036, 15482072

[bib2] Anderson, J. R., & Lebiere, C. (1998). The atomic components of thought. Erlbaum.

[bib3] Antoniou, K., Cummins, C., & Katsos, N. (2016). Why only some adults reject under-informative utterances. Journal of Pragmatics, 99, 78–95. 10.1016/j.pragma.2016.05.001

[bib4] Arslan, B., Taatgen, N. A., & Verbrugge, R. (2017). Five-year-olds’ systematic errors in second-order false belief tasks are due to first-order theory of mind strategy selection: A computational modeling study. Frontiers in Psychology, 8, 275. 10.3389/fpsyg.2017.00275, 28293206 PMC5329038

[bib5] Baker, C. L., Saxe, R., & Tenenbaum, J. B. (2009). Action understanding as inverse planning. Cognition, 113(3), 329–349. 10.1016/j.cognition.2009.07.005, 19729154

[bib6] Baron-Cohen, S., Wheelwright, S., Hill, J., Raste, Y., & Plumb, I. (2001). The “Reading the mind in the eyes” test revised version: A study with normal adults, and adults with Asperger syndrome or high-functioning autism. Journal of Child Psychology and Psychiatry, 42(2), 241–251. 10.1017/S0021963001006643, 11280420

[bib7] Barto, A. G. (1994). Adaptive critics and the basal ganglia. In J. C. Houk, J. L. Davis, & D. G. Beiser (Eds.), Models of information processing in the basal ganglia (pp. 215–232). MIT Press. 10.7551/mitpress/4708.001.0001

[bib8] Bilker, W. B., Hansen, J. A., Brensinger, C. M., Richard, J., Gur, R. E., & Gur, R. C. (2012). Development of abbreviated nineitem forms of the Raven’s standard progressive matrices test. Assessment, 19(3), 354–369. 10.1177/1073191112446655, 22605785 PMC4410094

[bib9] Bott, L., & Noveck, I. A. (2004). Some utterances are underinformative: The onset and time course of scalar inferences. Journal of Memory and Language, 51(3), 437–457. 10.1016/j.jml.2004.05.006

[bib10] Brasoveanu, A., & Dotlačil, J. (2020). Computational cognitive modeling and linguistic theory. Springer. 10.1007/978-3-030-31846-8

[bib11] Brasoveanu, A., & Dotlačil, J. (2021). Reinforcement learning for production-based cognitive models. Topics in Cognitive Science, 13, 467–487. 10.1111/tops.12546, 34105872

[bib12] Brennan, S. E., & Clark, H. H. (1996). Conceptual pacts and lexical choice in conversation. Journal of Experimental Psychology: Learning, Memory, and Cognition, 22(6), 1482–1493. 10.1037/0278-7393.22.6.1482, 8921603

[bib13] Bürkner, P.-C. (2017). Brms: An R package for Bayesian multilevel models using Stan. Journal of Statistical Software, 80(1), 1–28. 10.18637/jss.v080.i01

[bib14] Carpenter, P. A., Just, M. A., & Shell, P. (1990). What one intelligence test measures: A theoretical account of the processing in the Raven progressive matrices test. Psychological Review, 97(3), 404–431. 10.1037/0033-295x.97.3.404, 2381998

[bib15] Carstensen, A., Kon, E., & Regier, T. (2014). Testing a rational account of pragmatic reasoning: The case of spatial language. In Proceedings of the Annual Meeting of the Cognitive Science Society (Vol. 36, pp. 2009–2013).

[bib16] Ceballos, J. M., Stocco, A., & Prat, C. S. (2020). The role of basal ganglia reinforcement learning in lexical ambiguity resolution. Topics in Cognitive Science, 12(1), 402–416. 10.1111/tops.12488, 32023006

[bib17] Cheyette, S. J., & Piantadosi, S. T. (2024). Response to difficulty drives variation in IQ test performance. Open Mind: Discoveries in Cognitive Science, 8, 265–277. 10.1162/opmi_a_00127, 38571527 PMC10990577

[bib18] Cohen, M. X., & Ranganath, C. (2007). Reinforcement learning signals predict future decisions. Journal of Neuroscience, 27(2), 371–378. 10.1523/JNEUROSCI.4421-06.2007, 17215398 PMC6672075

[bib19] Cole, J. R., & Reitter, D. (2019). The role of working memory in syntactic sentence realization: A modeling & simulation approach. Cognitive Systems Research, 55, 95–106. 10.1016/j.cogsys.2019.01.001

[bib20] Cummins, C. (2025). Noncooperative communication. Annual Review of Linguistics, 11(1), 35–52. 10.1146/annurev-linguistics-011724-121451

[bib21] Dale, G., Sampers, D., Loo, S., & Green, C. S. (2018). Individual differences in exploration and persistence: Grit and beliefs about ability and reward. PLoS One, 13(9), e0203131. 10.1371/journal.pone.0203131, 30180200 PMC6122809

[bib22] Dang, J., King, K. M., & Inzlicht, M. (2020). Why are self-report and behavioral measures weakly correlated? Trends in Cognitive Sciences, 24(4), 267–269. 10.1016/j.tics.2020.01.007, 32160564 PMC7977810

[bib23] Degen, J. (2023). The rational speech act framework. Annual Review of Linguistics, 9(1), 519–540. 10.1146/annurev-linguistics-031220-010811

[bib24] Degen, J., & Franke, M. (2012). Optimal reasoning about referential expressions. In Proceedings of SemDial (Vol. 16, pp. 2–11).

[bib25] Dodell-Feder, D., Lincoln, S. H., Coulson, J. P., & Hooker, C. I. (2013). Using fiction to assess mental state understanding: A new task for assessing theory of mind in adults. PLoS One, 8(11), e81279. 10.1371/journal.pone.0081279, 24244736 PMC3820595

[bib26] Dotlačil, J. (2021). Parsing as a cue-based retrieval model. Cognitive Science, 45(8), e13020. 10.1111/cogs.13020, 34379334 PMC8459291

[bib27] Duckworth, A. L., & Quinn, P. D. (2009). Development and validation of the short grit scale (grit-S). Journal of Personality Assessment, 91(2), 166–174. 10.1080/00223890802634290, 19205937

[bib28] Duff, J., Mayn, A., & Demberg, V. (2025). An ACT-R model of resource-rational performance in a pragmatic reference game. In Proceedings of the Annual Meeting of the Cognitive Science Society (Vol. 47, pp. 840–847).

[bib29] Eisenberger, R., & Leonard, J. M. (1980). Effects of conceptual task difficulty on generalized persistence. American Journal of Psychology, 93(2), 285–298. 10.2307/1422233, 7406069

[bib30] Fairchild, S., & Papafragou, A. (2021). The role of executive function and theory of mind in pragmatic computations. Cognitive Science, 45(2), e12938. 10.1111/cogs.12938, 33616218

[bib31] Frank, M. C., Emilsson, A. G., Peloquin, B., Goodman, N. D., & Potts, C. (2016). Rational speech act models of pragmatic reasoning in reference games. PsyArXiv. 10.31234/osf.io/f9y6b_v1

[bib32] Frank, M. C., & Goodman, N. D. (2012). Predicting pragmatic reasoning in language games. Science, 336(6084), 998. 10.1126/science.1218633, 22628647

[bib33] Frank, M. J., Moustafa, A. A., Haughey, H. M., Curran, T., & Hutchison, K. E. (2007). Genetic triple dissociation reveals multiple roles for dopamine in reinforcement learning. Proceedings of the National Academy of Sciences, 104(41), 16311–16316. 10.1073/pnas.0706111104, 17913879 PMC2042203

[bib34] Frank, M. J., Seeberger, L. C., & O’Reilly, R. C. (2004). By carrot or by stick: Cognitive reinforcement learning in parkinsonism. Science, 306(5703), 1940–1943. 10.1126/science.1102941, 15528409

[bib35] Frank, M. J., Woroch, B. S., & Curran, T. (2005). Error-related negativity predicts reinforcement learning and conflict biases. Neuron, 47(4), 495–501. 10.1016/j.neuron.2005.06.020, 16102533

[bib36] Franke, M. (2011). Quantity implicatures, exhaustive interpretation, and rational conversation. Semantics & Pragmatics, 4, 1. 10.3765/sp.4.1

[bib37] Franke, M., & Degen, J. (2016). Reasoning in reference games: Individual- vs. population-level probabilistic modeling. PLoS One, 11(5), e0154854. 10.1371/journal.pone.0154854, 27149675 PMC4858259

[bib38] Frederick, S. (2005). Cognitive reflection and decision making. Journal of Economic Perspectives, 19(4), 25–42. 10.1257/089533005775196732

[bib39] Fu, W.-T., & Anderson, J. R. (2006). From recurrent choice to skill learning: A reinforcement-learning model. Journal of Experimental Psychology: General, 135(2), 184–206. 10.1037/0096-3445.135.2.184, 16719650

[bib40] Gonthier, C., Harma, K., & Gavornikova-Baligand, Z. (2024). Development of reasoning performance in Raven’s matrices is grounded in the development of effective strategy use. Journal of Experimental Psychology: General, 53(3), 689–705. 10.1037/xge0001518, 38059964

[bib41] Gonthier, C., & Thomassin, N. (2015). Strategy use fully mediates the relationship between working memory capacity and performance on Raven’s matrices. Journal of Experimental Psychology: General, 144(5), 916–924. 10.1037/xge0000101, 26413890

[bib42] Goodman, N. D., & Frank, M. C. (2016). Pragmatic language interpretation as probabilistic inference. Trends in Cognitive Sciences, 20(11), 818–829. 10.1016/j.tics.2016.08.005, 27692852

[bib43] Gray, W. D., Sims, C. R., Fu, W.-T., & Schoelles, M. J. (2006). The soft constraints hypothesis: A rational analysis approach to resource allocation for interactive behavior. Psychological Review, 113(3), 461–482. 10.1037/0033-295X.113.3.461, 16802878

[bib44] Grice, H. P. (1975). Logic and conversation. In P. Cole & J. L. Morgan (Eds.), Syntax and semantics (Vol. 3, pp. 41–58). Academic Press. 10.1163/9789004368811_003

[bib45] Hawkins, R. D., Gweon, H., & Goodman, N. D. (2021). The division of labor in communication: Speakers help listeners account for asymmetries in visual perspective. Cognitive Science, 45(3), e12926. 10.1111/cogs.12926, 33686646

[bib46] Hayes, T. R., Petrov, A. A., & Sederberg, P. B. (2011). A novel method for analyzing sequential eye movements reveals strategic influence on Raven’s advanced progressive matrices. Journal of Vision, 11(10), 10. 10.1167/11.10.10, 21926182

[bib47] Hendriks, P. (2016). Cognitive modeling of individual variation in reference production and comprehension. Frontiers in Psychology, 7, 506. 10.3389/fpsyg.2016.00506 27092101 PMC4823268

[bib48] Heyman, T., & Schaeken, W. (2015). Some differences in some: Examining variability in the interpretation of scalars using latent class analysis. Psychologica Belgica, 55(1), 1–18. 10.5334/pb.bc, 30479413 PMC5853968

[bib49] Hilbert, S., Nakagawa, T. T., Puci, P., Zech, A., & Bühner, M. (2014). The digit span backwards task. European Journal of Psychological Assessment, 31(3), 174–180. 10.1027/1015-5759/a000223

[bib50] Holroyd, C. B., & Coles, M. G. H. (2002). The neural basis of human error processing: Reinforcement learning, dopamine, and the error-related negativity. Psychological Review, 109(4), 679–709. 10.1037//0033-295X.109.4.679, 12374324

[bib51] Howes, A., Lewis, R. L., & Vera, A. (2009). Rational adaptation under task and processing constraints. Psychological Review, 116(4), 717–751. 10.1037/a0017187, 19839682

[bib52] Jäger, G. (2010). Game-theoretical pragmatics. In J. F. A. K. van Benthem & A. Ter Meulen (Eds.), Handbook of logic and language (pp. 467–491). Elsevier. 10.1016/B978-0-444-53726-3.00009-8,

[bib53] Jarosz, A. F., & Wiley, J. (2012). Why does working memory capacity predict RAPM performance? A possible role of distraction. Intelligence, 40(5), 427–438. 10.1016/j.intell.2012.06.001

[bib54] Kay, M. (2023). Extracting and visualizing tidy residuals from Bayesian models. Included with documentation for the tidybayes package.

[bib55] Kravtchenko, E., & Demberg, V. (2022). Informationally redundant utterances elicit pragmatic inferences. Cognition, 225, 105159. 10.1016/j.cognition.2022.105159, 35580451

[bib56] Krueger, P. M., Callaway, F., Gul, S., Griffiths, T. L., & Lieder, F. (2024). Identifying resource-rational heuristics for risky choice. Psychological Review, 131(4), 905–951. 10.1037/rev0000456, 38635156

[bib57] Lewis, R. L., & Vasishth, S. (2005). An activation-based model of sentence processing as skilled memory retrieval. Cognitive Science, 29, 375–419. 10.1207/s15516709cog0000_25, 21702779

[bib58] Lieder, F., & Griffiths, T. L. (2020). Resource-rational analysis: Understanding human cognition as the optimal use of limited computational resources. Behavioral and Brain Sciences, 43, e1. 10.1017/S0140525X1900061X, 30714890

[bib59] Mayn, A., & Demberg, V. (2023). High performance on a pragmatic task may not be the result of successful reasoning: On the importance of eliciting participants’ reasoning strategies. Open Mind: Discoveries in Cognitive Science, 7, 156–178. 10.1162/opmi_a_00077, 37416077 PMC10320817

[bib60] Mayn, A., & Demberg, V. (2026). Sources of individual variability in a pragmatic reference game: Effects of logical reasoning and theory of mind. PLoS One, 21(2), e0339899. 10.1371/journal.pone.0339899, 41712667 PMC12919809

[bib61] Misyak, J., Noguchi, T., & Chater, N. (2016). Instantaneous conventions: The emergence of flexible communicative signals. Psychological Science, 27(12), 1550–1561. 10.1177/0956797616661199, 27793986 PMC5221723

[bib62] Nicenboim, B., Logačev, P., Gattei, C., & Vasishth, S. (2016). When high-capacity readers slow down and low-capacity readers speed up: Working memory and locality effects. Frontiers in Psychology, 7, 280. 10.3389/fpsyg.2016.00280, 27014113 PMC4782223

[bib63] Parikh, P. (1991). Communication and strategic inference. Linguistics and Philosophy, 14(5), 473–514. 10.1007/bf00632595

[bib64] Patil, U., & Lago, S. (2021). Prediction advantage as retrieval interference: An ACT-R model of processing possessive pronouns. In Proceedings of the 19th International Conference on Cognitive Modeling (pp. 213–219). 10.31219/osf.io/9vwa3

[bib65] Qing, C., & Franke, M. (2015). Variations on a Bayesian theme: Comparing Bayesian models of referential reasoning. In H. Zeevat & H.-C. Schmitz (Eds.), Bayesian natural language semantics and pragmatics (pp. 201–220). Springer. 10.1007/978-3-319-17064-0_9

[bib66] Raven, J., & Raven, J. (2003). Raven progressive matrices. In R. S. McCallum (Ed.), Handbook of nonverbal assessment (pp. 223–237). Springer. 10.1007/978-1-4615-0153-4_11

[bib67] Raven, J., Raven, J. C., & Court, J. H. (1998). Manual for Raven’s progressive matrices and vocabulary scale. Oxford Psychologists Press.

[bib68] Reitter, D., Keller, F., & Moore, J. D. (2011). A computational cognitive model of syntactic priming. Cognitive Science, 35(4), 587–637. 10.1111/j.1551-6709.2010.01165.x, 21564266

[bib69] Rohde, H., Seyfarth, S., Clark, B., Jäger, G., & Kaufmann, S. (2012). Communicating with cost-based implicature: A game-theoretic approach to ambiguity. In Proceedings of SemDial 2012 (SeineDial): The 16th Workshop on Semantics and Pragmatics of Dialogue (pp. 107–116).

[bib70] Ryzhova, M., Mayn, A., & Demberg, V. (2023). What inferences do people actually make upon encountering informationally redundant utterances? An individual differences study. In Proceedings of the Annual Meeting of the Cognitive Science Society (Vol. 45, pp. 2631–2638).

[bib71] Shipstead, Z., Harrison, T. L., & Engle, R. W. (2016). Working memory capacity and fluid intelligence: Maintenance and disengagement. Perspectives on Psychological Science, 11(6), 771–799. 10.1177/1745691616650647, 27899724

[bib72] Sikos, L., Venhuizen, N. J., Drenhaus, H., & Crocker, M. W. (2021a). Reevaluating pragmatic reasoning in language games. PLOS One, 16(3), e0248388. 10.1371/journal.pone.0248388, 33730097 PMC7968720

[bib73] Sikos, L., Venhuizen, N. J., Drenhaus, H., & Crocker, M. W. (2021b). Speak before you listen: Pragmatic reasoning in multitrial language games. In Proceedings of the Annual Meeting of the Cognitive Science Society (Vol. 43, pp. 1465–1471).

[bib74] Stiller, A. J., Goodman, N. D., & Frank, M. C. (2011). Ad-hoc scalar implicature in adults and children. In Proceedings of the Annual Meeting of the Cognitive Science Society (Vol. 33, pp. 2134–2139).

[bib75] Stiller, A. J., Goodman, N. D., & Frank, M. C. (2015). Ad-hoc implicature in preschool children. Language Learning and Development, 11(2), 176–190. 10.1080/15475441.2014.927328

[bib76] Stocco, A. (2018). A biologically plausible action selection system for cognitive architectures: Implications of basal ganglia anatomy for learning and decision-making models. Cognitive Science, 42, 457–490. 10.1111/cogs.12506, 28585747

[bib77] Stocco, A., Murray, N. L., Yamasaki, B. L., Renno, T. J., Nguyen, J., & Prat, C. S. (2017). Individual differences in the Simon effect are underpinned by differences in the competitive dynamics in the basal ganglia: An experimental verification and a computational model. Cognition, 164, 31–45. 10.1016/j.cognition.2017.03.001, 28363106

[bib78] Stocco, A., Prat, C. S., & Graham, L. K. (2021). Individual differences in reward-based learning predict fluid reasoning abilities. Cognitive Science, 45, e12941. 10.1111/cogs.12941, 33619738

[bib79] Storm, B. C., Angello, G., & Bjork, E. L. (2011). Thinking can cause forgetting: Memory dynamics in creative problem solving. Journal of Experimental Psychology: Learning, Memory, and Cognition, 37(5), 1287–1293. 10.1037/a0023921, 21707211

[bib80] Sutton, R. S., & Barto, A. G. (2018). Reinforcement learning: An introduction (2nd ed.). MIT Press.

[bib81] Taatgen, N. A. (2002). A model of individual differences in skill acquisition in the Kanfer-Ackerman air traffic control task. Cognitive Systems Research, 3, 103–112. 10.1016/S1389-0417(01)00049-3

[bib82] Taatgen, N. A. (2013). The nature and transfer of cognitive skills. Psychological Review, 120(3), 439–471. 10.1037/a0033138, 23750831

[bib83] Taatgen, N. A., & Anderson, J. R. (2002). Why do children learn to say “broke”? A model of learning the past tense without feedback. Cognition, 86, 123–155. 10.1016/S0010-0277(02)00176-2, 12435534

[bib84] Taatgen, N. A., & Lee, F. J. (2003). Production compilation: A simple mechanism to model complex skill acquisition. Human Factors, 45(1), 61–76. 10.1518/hfes.45.1.61.27224, 12916582

[bib85] Taatgen, N. A., van Rijn, H., & Anderson, J. (2007). An integrated theory of prospective time interval estimation: The role of cognition, attention, and learning. Psychological Review, 114(3), 577–598. 10.1037/0033-295X.114.3.577, 17638497

[bib86] Thissen, D. (1983). Timed testing: An approach using item response theory. In New horizons in testing: Latent trait test theory and computerized adaptive testing (pp. 179–203). Academic Press. 10.1016/B978-0-12-742780-5.50019-6

[bib87] Trott, S., & Bergen, B. (2019). Individual differences in mentalizing capacity predict indirect request comprehension. Discourse Processes, 56(8), 675–707. 10.1080/0163853X.2018.1548219

[bib88] Trott, S., & Bergen, B. (2020). When do comprehenders mentalize for pragmatic inference? Discourse Processes, 57(10), 900–920. 10.1080/0163853X.2020.1822709

[bib89] Unsworth, N., Heitz, R. P., Schrock, J. C., & Engle, R. W. (2005). An automated version of the operation span task. Behavior Research Methods, 37(3), 498–505. 10.3758/BF03192720, 16405146

[bib90] Van Rooy, R. (2004). Signalling games select horn strategies. Linguistics and Philosophy, 27, 493–527. 10.1023/B:LING.0000024403.88733.3f

[bib91] Vehtari, A., Gelman, A., & Gabry, J. (2017). Practical Bayesian model evaluation using leave-one-out cross-validation and WAIC. Statistics and Computing, 27(5), 1413–1432. 10.1007/s11222-016-9696-4

[bib92] Ventura, M., & Shute, V. (2013). The validity of a game-based assessment of persistence. Computers in Human Behavior, 29(6), 2568–2572. 10.1016/j.chb.2013.06.033

[bib93] Vigneau, F., Caissie, A. F., & Bors, D. A. (2006). Eye-movement analysis demonstrates strategic influences on intelligence. Intelligence, 34(3), 261–272. 10.1016/j.intell.2005.11.003

[bib94] von Bastian, C. C., Belleville, S., Udale, R. C., Reinhartz, A., Essounni, M., & Strobach, T. (2022). Mechanisms underlying training-induced cognitive change. Nature Reviews Psychology, 1(1), 30–41. 10.1038/s44159-021-00001-3 PMC9270821 35804410

[bib95] Yang, Y. C., Karmol, A. M., & Stocco, A. (2021). Core cognitive mechanisms underlying syntactic priming: A comparison of three alternative models. Frontiers in Psychology, 12, 662345. 10.3389/fpsyg.2021.662345, 34262508 PMC8273879

